# Evaluation of the Biochemical, Colorimetric, and Microbiological Stability of Red Cabbage Anthocyanin Extracts for Application as Natural Indicators

**DOI:** 10.3390/molecules31183339

**Published:** 2026-09-20

**Authors:** Loredana-Mariana Huțuțui, Sonia Amariei, Anca-Mihaela Gâtlan

**Affiliations:** Faculty of Food Engineering, Ștefan cel Mare University of Suceava, 720229 Suceava, Romania; loredana.hututui@student.usv.ro (L.-M.H.); anca.gatlan@fia.usv.ro (A.-M.G.)

**Keywords:** anthocyanins, antioxidant activity, color stability, degradation kinetics, red cabbage

## Abstract

Food spoilage is accompanied by changes in pH, which allows freshness to be monitored using colorimetric indicators. Due to their color response, the anthocyanins in red cabbage are of interest for smart packaging. However, their applicability depends on their stability during storage. This study evaluated the stability of red cabbage anthocyanin extracts obtained in water and in 50% ethanol, monitored for 10 days by analyzing total anthocyanin content (TAC), antioxidant activity (DPPH), colorimetric properties, color stability as a function of pH, degradation kinetics, and microbiological load. The ethanol extracts exhibited the best stability, with TAC decreasing from 116 to 73 mg CGE/100 g FW (t½ = 16–76 days), compared to the aqueous extracts, in which TAC decreased from 74 to 38 mg CGE/100 g FW (t½ = 9.5 days). DPPH followed the same trend, with a maximum half-life (t½) of 67.7 days. Temperature was the main factor contributing to degradation, followed by light and oxygen. Color varied depending on pH, ranging from red under acidic conditions to blue–green under alkaline conditions. The ethanolic extract exhibited the best color stability (ΔE < 1). Its stability at 4 °C, corresponding to food refrigeration conditions, highlights its potential as a colorimetric indicator.

## 1. Introduction

In the food industry, packaging plays an essential role in maintaining food quality and safety and in extending its shelf life [1,2]. During storage, distribution, and transportation, food products may undergo changes that affect their quality and safety. In general, consumers assess the quality and freshness of packaged foods based on the expiration date printed on the packaging. However, for some perishable products, the expiration date does not always reflect the product’s actual condition [1]. To maintain quality standards, the food industry needs a fast and sustainable method for monitoring and evaluating food products. In this context, the use of smart packaging—capable of providing real-time information on food freshness—offers an innovative and practical solution for the food industry [3,4,5]. Smart packaging can utilize pH monitoring systems that provide rapid and accurate visual information on changes in food quality, enabling the monitoring of products during transport and storage [6,7,8,9].

Anthocyanins have attracted considerable interest as natural pigments for the development of pH-sensitive smart packaging. Red cabbage (*Brassica oleracea* L. var. *capitata* f. *rubra*), a species belonging to the Brassicaceae family and widely cultivated due to its adaptability and high productivity, is one of the most important plant sources of anthocyanins, along with other bioactive compounds such as vitamin C, glucosinolates, carotenoids, phenolic acids, and flavonols [10]. Due to its high pigment content and color stability, red cabbage is frequently used as a source of natural dyes and as a raw material for the production of colorimetric indicators used in food quality monitoring [11,12]. In addition to its colorimetric characteristics, red cabbage is of particular interest due to its high content of phenolic compounds and anthocyanins, which are associated with strong antioxidant activity. These compounds may help limit the oxidative reactions responsible for the degradation of lipids, pigments, and other food constituents, thereby helping to maintain food quality during storage [13,14]. In this context, the combination of favorable antioxidant activity with good microbiological stability of the extracts may be an advantage for their use as natural sources of bioactive compounds with preservative potential.

Anthocyanins are water-soluble polyphenolic compounds from the flavonoid class, known for their antioxidant properties [1,15]. Structurally, they are glycosidic forms of anthocyanidins and are based on the characteristic C6–C3–C6 skeleton of the flavylium cation. An important characteristic that supports the use of anthocyanins in indicator applications is their sensitivity to pH variations, reflected in reversible changes in structure and color. Thus, anthocyanins are predominantly found as the flavylium cation (red) at pH 1–3, as the carbinol pseudobase (nearly colorless) at pH 4–5, the quinonoid base (violet) at pH 6–7, the anionic form of the quinonoid base (blue) at pH 7–8, and as chalcone (yellow) in an alkaline medium (pH > 8) [16,17,18].

Red cabbage extract contains over 30 anthocyanins, predominantly acylated derivatives of cyanidin, which exhibit superior thermal and photochemical stability and a broader color range compared to non-acylated forms—characteristics that make them well-suited for use in smart food freshness-monitoring systems [11,12].

However, anthocyanins are relatively unstable compounds that are sensitive to oxygen, light, temperature, and water activity [19]. Prolonged exposure to these factors promotes oxidation, hydration, and degradation of the flavylium core, leading to color loss and a reduction in the concentration of bioactive compounds [20,21,22]. This instability may limit their applicability in smart packaging, as pigment degradation gradually reduces the ability to detect changes associated with food freshness [23]. Therefore, evaluating the stability of the extracts is an essential step prior to their integration into indicator systems designed to monitor food.

In this context, the aim of this study was to evaluate the stability of anthocyanin extracts from red cabbage and to highlight their potential for use as natural indicators integrated into smart packaging for monitoring food freshness.

The stability of the extracts obtained in water and in 50% ethanol, was analyzed under various storage conditions, varying temperature, light exposure, and oxygen presence. The evaluation was performed by determining the total anthocyanin content (TAC), antioxidant activity using the DPPH method, and microbial load. To describe the behavior of anthocyanins and antioxidant activity over time, the degradation rate constant (k) and half-life (t^1/2^) were determined. In addition, colorimetric parameters from the CIELAB system (L*, a*, b*, C*, h, and ΔE) were determined, as well as spectrophotometric parameters associated with pigment degradation, namely color density (CD), polymeric color (PC), browning index (BI), and degradation index (DI). At the same time, the evolution of the extracts’ color as a function of pH during storage was monitored to evaluate the maintenance of the chromatic response necessary for their function as freshness indicators.

Although the properties of anthocyanins in red cabbage and their stability as a function of pH and temperature have been investigated in previous studies, the comparative and integrated evaluation of the influence of the extraction solvent, temperature, light, oxygen, and storage time on the stability of the extracts has been less explored. In this context, the novelty of the present study lies in the simultaneous evaluation of these factors using a full factorial design, which allows for the identification of both individual effects and interactions among the experimental factors. Furthermore, the comparison of aqueous and ethanolic extracts under controlled conditions, the monitoring of changes in microbial load during storage, and the evaluation of the chromatic response over the pH range of 1–10 provide an integrated characterization of the stability and functional potential of the extracts.

## 2. Results

### 2.1. Physicochemical Characterization of Red Cabbage

Analysis of the physicochemical parameters (Table 1) revealed that red cabbage has a high moisture content (91.72 ± 0.74%) and a water activity of 0.98 ± 0.002, characteristics specific to fresh raw materials that can influence microbiological stability. High moisture and water activity values promote degradation processes during storage, which is why they must be taken into account when evaluating the stability of the extracts. The low pro-tein content (1.01 ± 0.02 g/100 g FW) and lipid content (0.10 ± 0.01 g/100 g FW) contribute to maintaining stability, as these components are involved in oxidative processes that can accelerate the degradation of bioactive compounds and color changes during storage [24,25]. The carbohydrate content, calculated by difference (5.90 g/100 g FW), and the crude fiber content (0.71 ± 0.02 g/100 g FW) may contribute to the stability of bioactive compounds through interactions with polyphenols and anthocyanins, thereby influencing the behavior of the extracts during storage [12]. The pH (6.17 ± 0.18) is an important parameter influencing the degradation of anthocyanins, as these pigments are more stable in acidic environments [26]. Therefore, the physicochemical characterization of the raw material provides essential information for interpreting the changes that may occur in the composition and stability of red cabbage extracts during storage.

### 2.2. The Effect of pH on the Color Parameters of Red Cabbage Extracts

**Figure 1:**
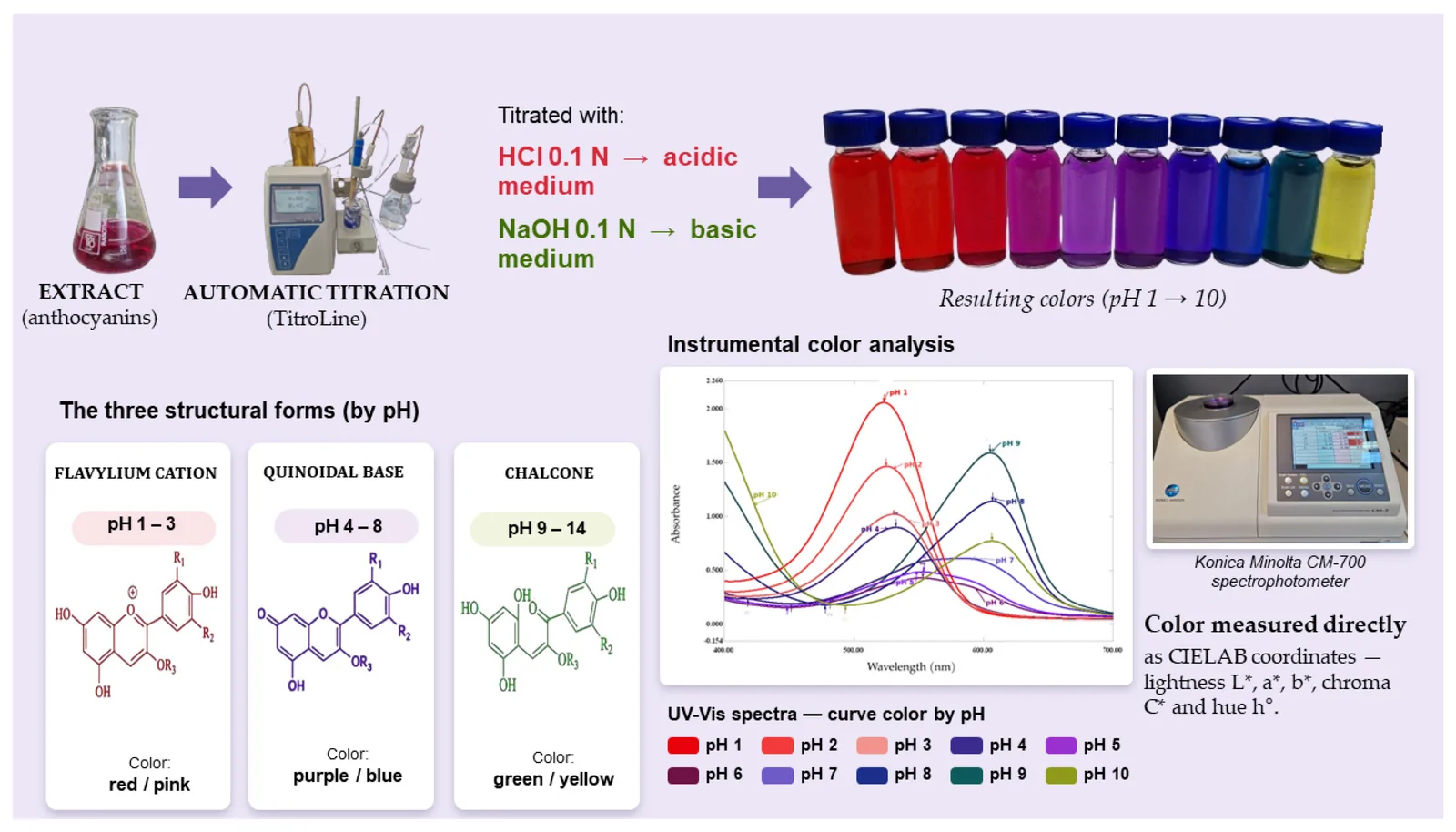
Chromatic and anthocyanin behavior in the pH range of 1–10, as determined by titration, UV-Vis spectrophotometry, and colorimetric analysis.

The stability and color of anthocyanins are closely linked to their chemical structure. The system of conjugated double bonds allows these pigments to absorb light at a wavelength of approximately 500 nm, producing shades of red, violet, blue, and intermediate tones, as shown in Figure 1 [26]. However, anthocyanins are highly unstable and easily degraded, being affected by pH, temperature, light, and oxygen [12]. The color parameters of red cabbage extracts obtained in 50% ethanol and in water (Table 2 and Table 3), showed a pronounced dependence on pH, a characteristic of anthocyanins.

At acidic pH (1–4), the a* coordinate had positive values (red component), the chroma C* was at its maximum, and the hue angle h fell within the red–purple range (305–350°), corresponding to the predominance of the flavylium cation. As the pH increased toward neutral (7–8), a* decreased toward zero or slightly negative values, b* remained negative, chroma decreased, and the hue shifted toward blue-violet (260–285°). At alkaline pH (9–10), a* became distinctly negative, chroma reached its minimum values, and hue shifted toward 210–250° (the blue-green range), reflecting the transition to quinoid bases and decolorized forms. Of the five coordinates, a* was the parameter most sensitive to pH variation, acting as the primary indicator of the transition from red to blue.

The a* parameter describes the variation in color along the green (−) to red (+) axis, with positive values indicating a predominance of red hues. In both extracts, the a* values were positive in the acidic and slightly acidic ranges (pH 1–7), confirming the predominance of red–violet hues characteristic of the flavyl cation in anthocyanins. The ethanolic extract exhibited higher a* values compared to the aqueous extract, particularly at acidic pH, where values of 2.96 ± 0.30 and 1.55 ± 0.13 were recorded at pH 1. As the pH increased, the a* values decreased progressively, becoming negative in the alkaline range (pH 9–10), indicating a reduction in the red component and the appearance of bluish-green hues associated with structural transformations of the anthocyanins.

The total color difference ΔE revealed significant changes between the two extracts. In the case of the 50% ethanolic extract, ΔE remained (< 1) throughout the entire storage period, except in the alkaline range, where it reached 2.13 at pH 9 on day 10, indicating a stable color over the 10 days. According to the NBS scale, for the 50% ethanolic extract, the values fell almost exclusively within the “trace” and “slight” levels throughout the entire storage period, indicating color differences that were imperceptible or barely perceptible to the naked eye.

A different behavior was observed for the aqueous extract, which exhibited much more pronounced color variations. In the weakly acidic–neutral range (pH 3–8), color changes were minimal during the first few days of storage but became more pronounced after day 5. At pH 6 and 7, NBS values reached 3.1–3.6, indicating clearly observable color differences, and at pH 5 they reached 6.4, reflecting significant color changes. On day 10, NBS values increased to 7.3–10.5 for pH 3, 5, 6, 7, and 8, and at pH 4 they reached a maximum of 12.66 (ΔE = 13.76), highlighting very pronounced color changes that were easily perceptible to the naked eye. In contrast, at strongly acidic (1–2) and alkaline (9–10) pH levels, the NBS values remained low, suggesting better color stability of the anthocyanins under these conditions.

### 2.3. Analysis of Colorimetric Indices (CD, PC, BI, DI)

#### 2.3.1. Color Density (CD)

Color density (CD) is used to assess the overall color intensity of anthocyanin-rich extracts and serves as a sensitive indicator of color changes that occur during storage [27,28]. Monitoring this parameter allows for the assessment of the preservation of natural pigments, as a decrease in color density is associated with the degradation of anthocyanins and a reduction in the visual intensity of the extract. During storage, CD values decreased in all samples (*p* < 0.001), indicating the progressive degradation of colored compounds.

**Figure 2:**
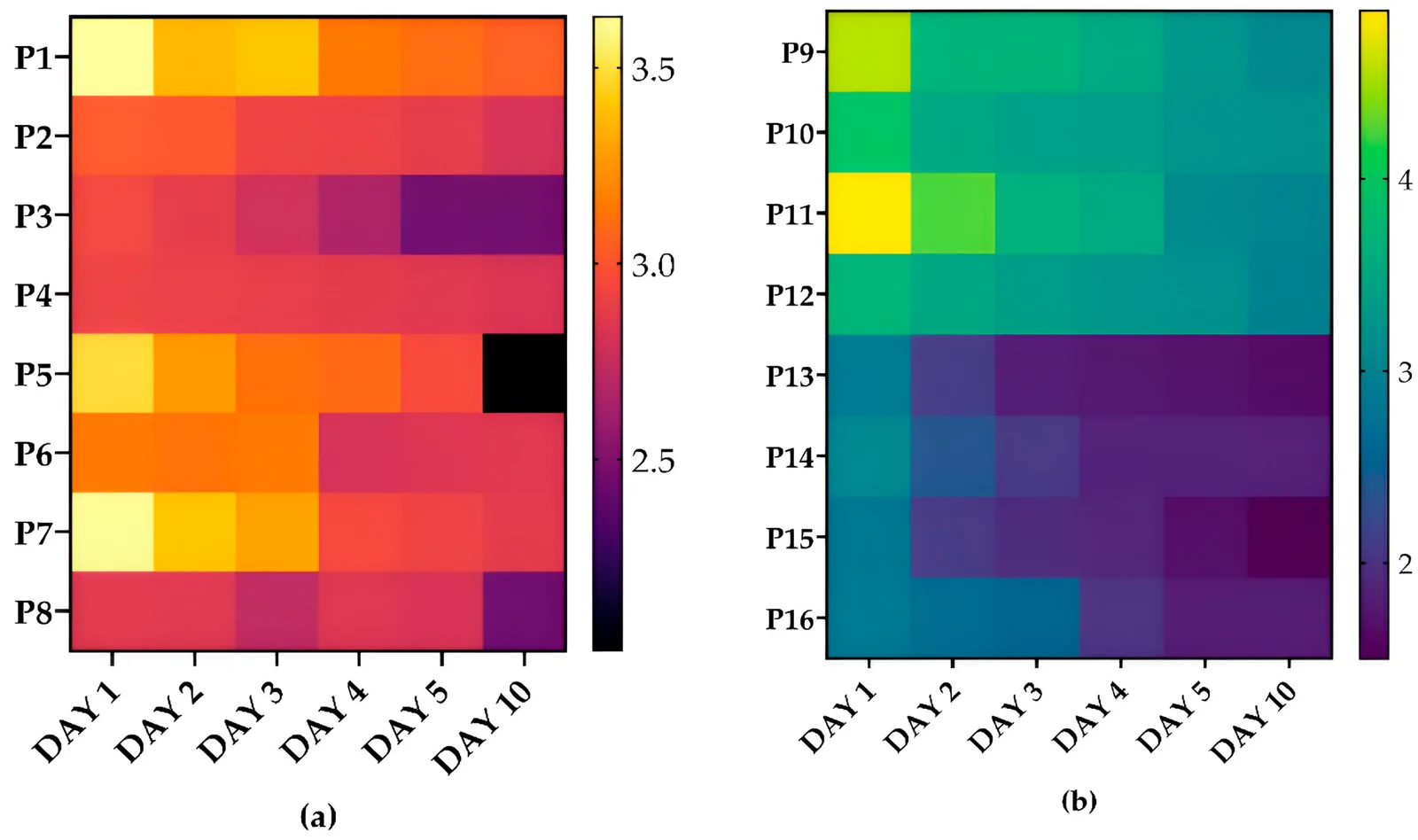
Changes in color density (CD) of ethanolic (**a**) and aqueous (**b**) extracts of red cabbage during storage.

In the ethanol extracts (Figure 2a), a decrease in color density was evident in all the samples analyzed. The greatest reduction was observed in sample P5, while sample P2 exhibited the best stability, with minimal variations between day 1 and day 10. In general, samples stored at 4 °C retained their color intensity more effectively, while samples exposed to light showed a more rapid decline (day × light: *p* = 0.021).

In the aqueous extracts (Figure 2b), the CD values were initially higher than in the ethanolic extracts, but they also showed a decreasing trend. The best stability was observed in sample P10, where the color density decreased from 3.920 to 3.170, while sample P11 showed the most pronounced decrease, from 4.870 to 2.980.

Temperature significantly influenced color density (*p* = 0.002) and interacted with the solvent (*p* = 0.001), with aqueous extracts being much more sensitive to temperature than ethanolic extracts. The rate of decline was significantly influenced by the solvent (day × solvent: *p* = 0.001), while oxygen had no significant effect, either as a main factor (*p* = 0.532) or in interaction with time (*p* = 0.704).

#### 2.3.2. Polymer Color (PC)

Polymer color (PC) is an indicator of the transformation of monomeric anthocyanins into more stable polymeric pigments, formed during storage through condensation and oxidation reactions [28]. This parameter allows for the assessment of the stability of plant extracts, as increasing PC values indicate the progression of anthocyanin degradation processes and the formation of compounds responsible for brown color. PC values increased in all samples during storage, indicating the progressive accumulation of these compounds.

**Figure 3:**
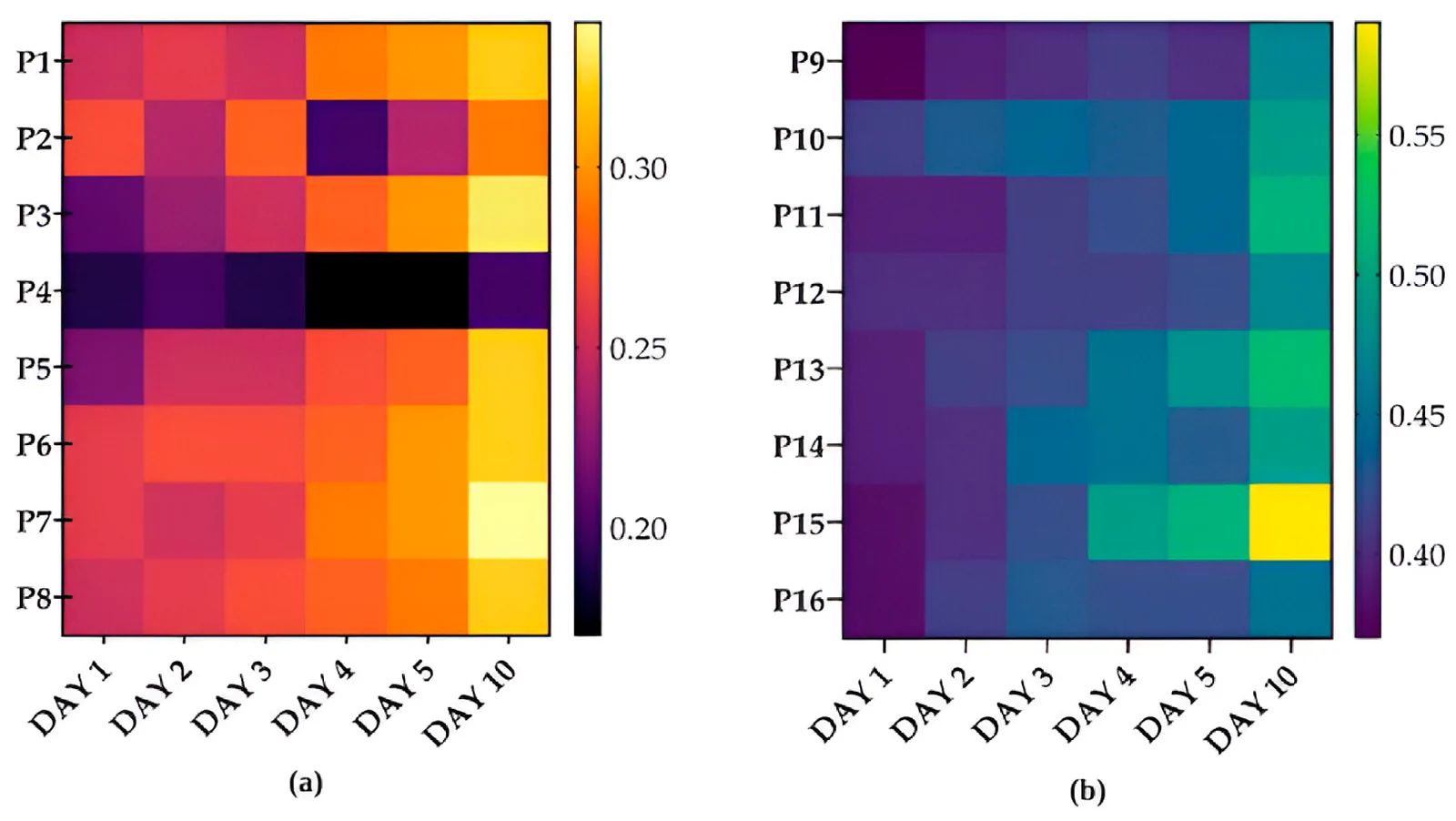
Changes in the polymeric color (PC) of the ethanolic (**a**) and aqueous (**b**) extracts of red cabbage during storage.

In the ethanol extracts (Figure 3a), the PC values showed moderate increases. Sample P3 showed the greatest variation, while sample P4 exhibited the best stability, with the PC increasing only slightly, from 0.19 on day 1 to 0.20 on day 10. Additionally, samples stored in the dark showed smaller variations compared to those exposed to light (day × light: *p* = 0.010).

In the aqueous extracts (Figure 3b), the PC values were higher than in the ethanolic extracts throughout the monitoring period. The best stability was observed in samples P12 and P16, where the values increased from 0.400 to 0.470 and from 0.380 to 0.450, respectively, while sample P13 showed the most pronounced increase, from 0.390 to 0.520.

Compared to the ethanolic extracts, the aqueous extracts showed higher PC values (*p* < 0.001), suggesting a more pronounced accumulation of polymeric pigments during storage. The accumulation rate of polymeric pigments was significantly influenced by light (*p* = 0.010), while temperature (*p* = 0.109) and oxygen (*p* = 0.564) had no significant effects.

#### 2.3.3. Degradation Index (DI)

The degradation index (DI) is one of the most relevant parameters used to assess the stability of anthocyanin-rich extracts, as it expresses the ratio between degraded pigments and those remaining in monomeric form [29,30]. Increasing DI values indicate intensified degradation processes and the progressive transformation of color-responsible compounds, serving as a direct indicator of stability loss during storage. DI values increased in all samples analyzed over the course of storage.

**Figure 4:**
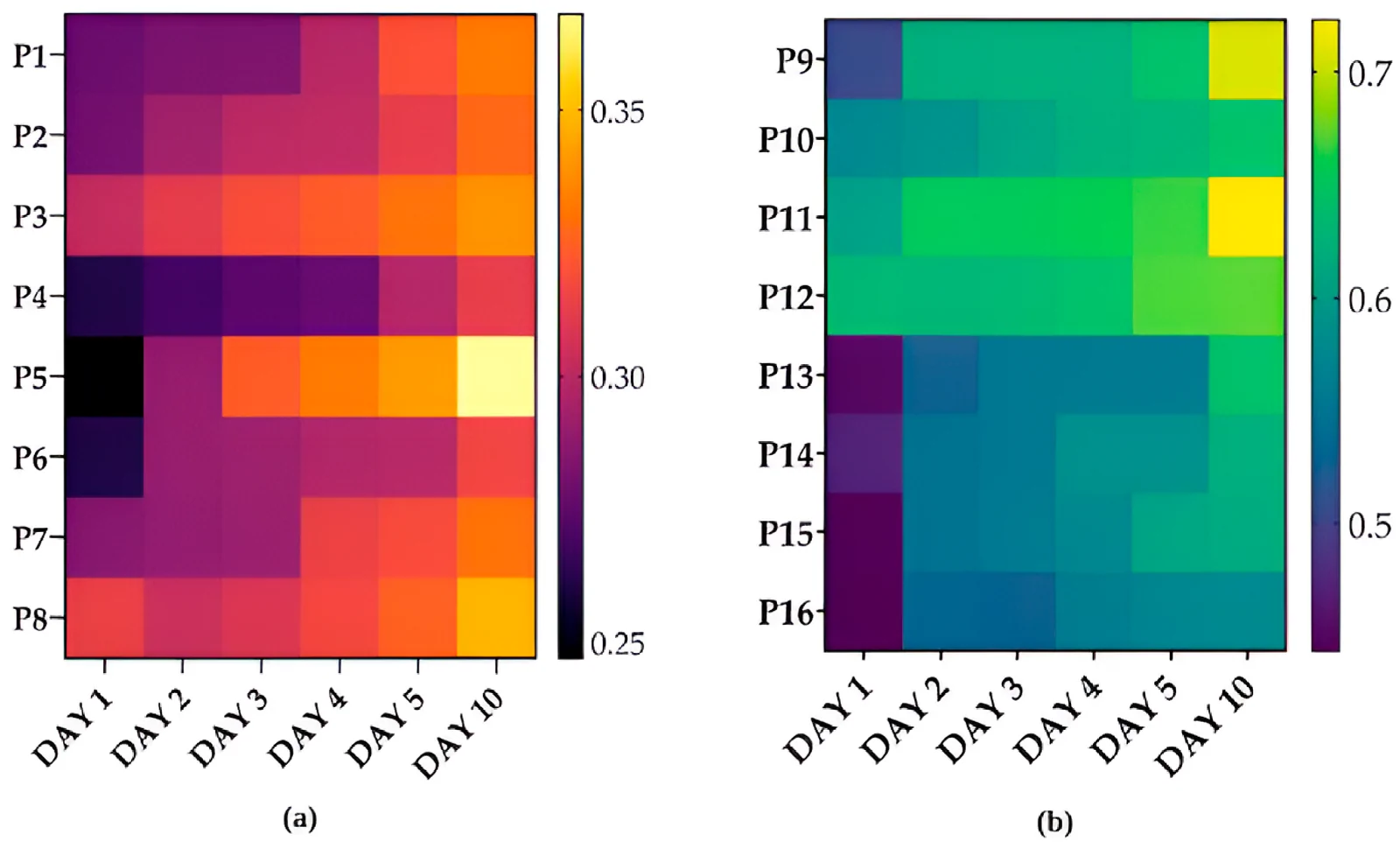
Changes in the degradation index (DI) of ethanolic (**a**) and aqueous (**b**) extracts of red cabbage during storage.

In the ethanol extracts (Figure 4a), the greatest increase was observed in sample P5, while sample P4 exhibited the best stability, with little variation between the beginning and end of the storage period.

In the aqueous extracts (Figure 4b), the best stability was observed in sample P12, where the degradation index increased from 0.630 to 0.674, while sample P9 showed the greatest increase, from 0.505 to 0.708.

The solvent significantly influenced the DI values (*p* < 0.001), with aqueous extracts showing significantly higher values than ethanolic extracts. Temperature also significantly influenced the DI values (*p* = 0.009) and interacted with the solvent (*p* = 0.004), with aqueous extracts being more sensitive to temperature. The rate of increase in the degradation index differed significantly between the two types of extracts (day × solvent: *p* = 0.005), while oxygen had no significant effect (*p* = 0.336).

#### 2.3.4. Browning Index (BI)

The Browning Index (BI) is one of the most widely used indicators of non-enzymatic browning reactions. Its increase reflects the accumulation of intermediate and final compounds of the Maillard reaction and is associated with changes in color, aroma, and stability [30,31]. The BI increased in all samples during storage, indicating the progressive accumulation of browning compounds.

**Figure 5:**
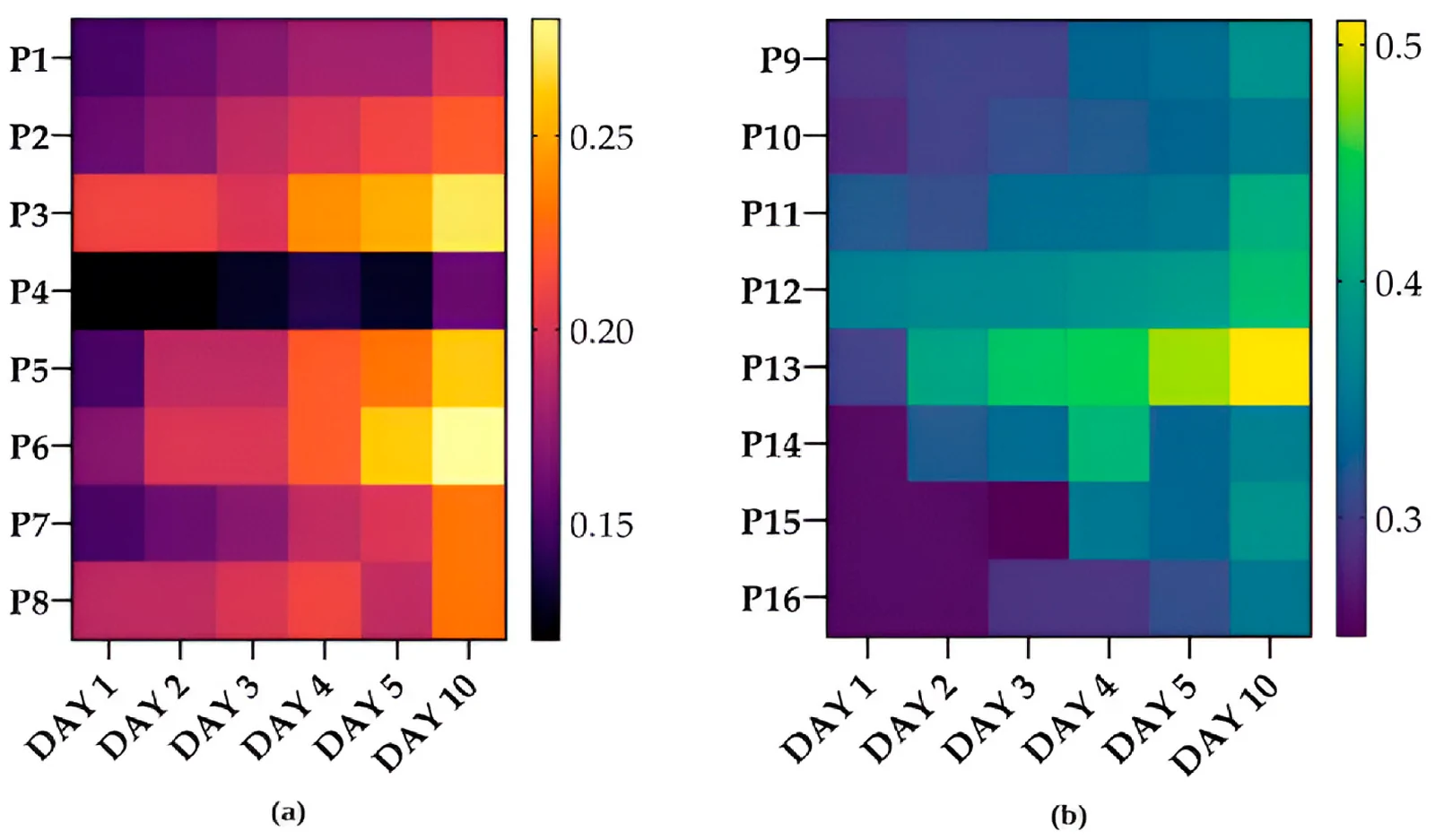
Changes in the browning index (BI) of ethanolic (**a**) and aqueous (**b**) extracts of red cabbage during storage.

In the ethanol extracts (Figure 5a), the BI values remained relatively low throughout the storage period. Sample P5, stored at 25 °C, showed the greatest increase in the browning index.

In the aqueous extracts (Figure 5b), the BI values were generally higher than in the ethanolic extracts. The best stability was observed in sample P12, where the browning index increased from 0.360 on day 1 to 0.430 on day 10, while sample P13, stored at 25 °C, showed the most pronounced variation, from 0.300 to 0.510.

The browning index increased significantly during storage (*p* < 0.001), with the solvent being the only factor with a significant influence (*p* = 0.002); the ethanol extracts showed values significantly lower than those of the aqueous extracts. Light (*p* = 0.575), temperature (*p* = 0.685), and oxygen (*p* = 0.554) did not significantly influence the BI values, either as main factors or in interaction with storage time, suggesting that non-enzymatic browning depends primarily on the nature of the extraction medium and the duration of storage.

### 2.4. Stability of Red Cabbage Extract

#### 2.4.1. Kinetic Parameters of Anthocyanin Degradation and Antioxidant Activity

**Figure 6:**
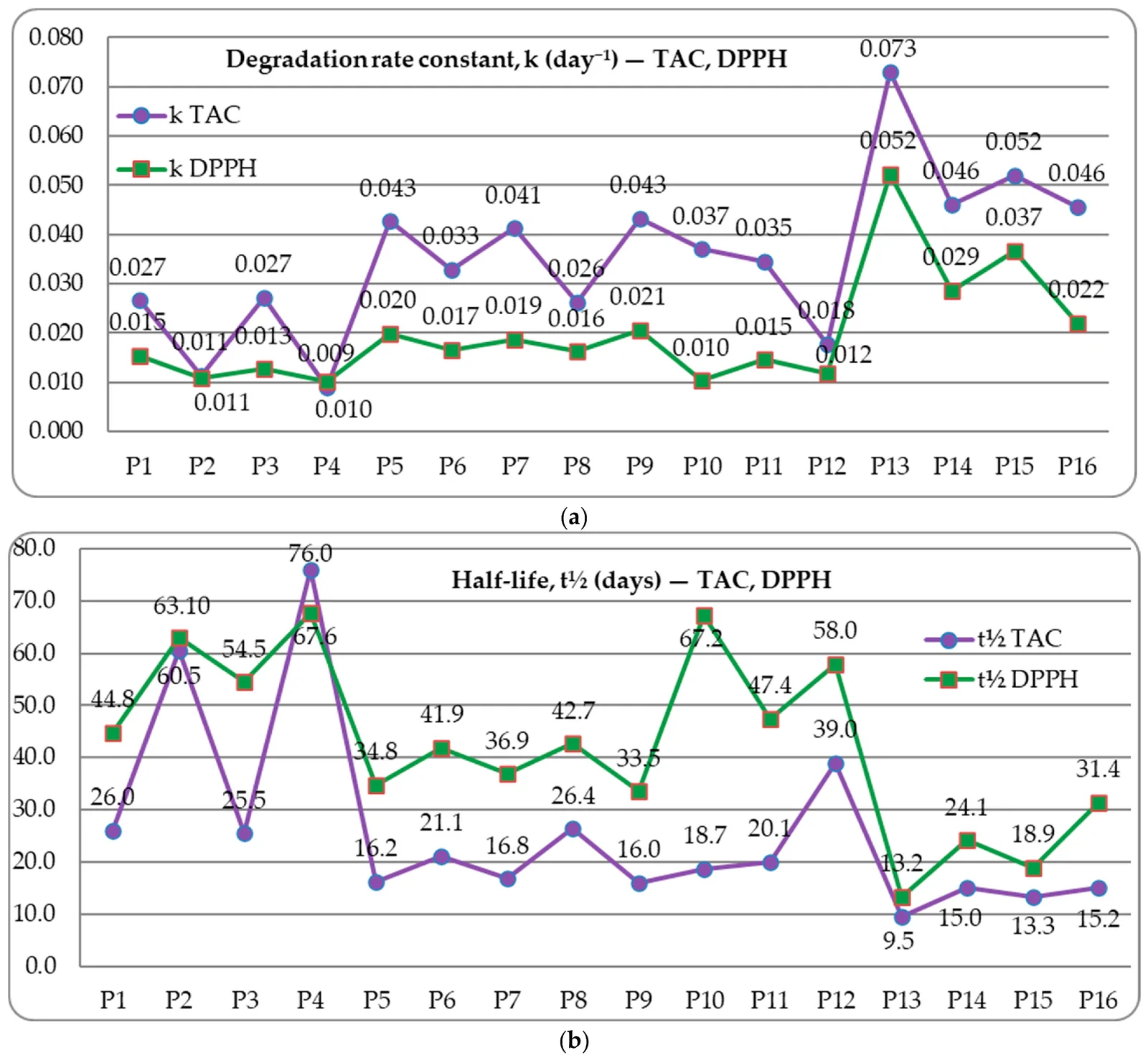
(**a**) Values of the degradation rate constant (k) for total anthocyanin content (TAC) and antioxidant activity (DPPH) of red cabbage extracts. (**b**) Half-life (t½) values for total anthocyanin content (TAC) and antioxidant activity (DPPH) of red cabbage extracts.

The changes in total anthocyanin content (TAC) and antioxidant activity (DPPH) over the 10-day storage period were described using a first-order kinetic model, according to Equations (12) and (13) presented in the Section 4. The stability of the extracts was evaluated by determining the degradation constant (k) and the half-life (t^1/2^), two parameters frequently used to characterize the rate of degradation and the persistence of bioactive compounds over time [21]. In general, an increase in temperature accelerates degradation processes, which is reflected in higher values of the constant k and a shorter half-life for both anthocyanins and the associated antioxidant activity [32]. These kinetic parameters were calculated for each of the 16 samples, and the resulting values are plotted in Figure 6.

#### 2.4.2. The Effect of Oxygen on Anthocyanin Content and Antioxidant Activity (DPPH)

Oxygen is one of the main factors influencing the stability of anthocyanins in red cabbage, due to the presence of unsaturated bonds and numerous phenolic hydroxyl groups in their structure, which make them highly susceptible to oxidative degradation [12]. The action of oxygen can accelerate the degradation of anthocyanins both through direct oxidative mechanisms affecting the flavylium structure and by stimulating the activity of endogenous oxidative enzymes, particularly polyphenol oxidase, which leads to progressive discoloration, reduced stability of the compounds, and loss of antioxidant and biological activity [20].

**Figure 7:**
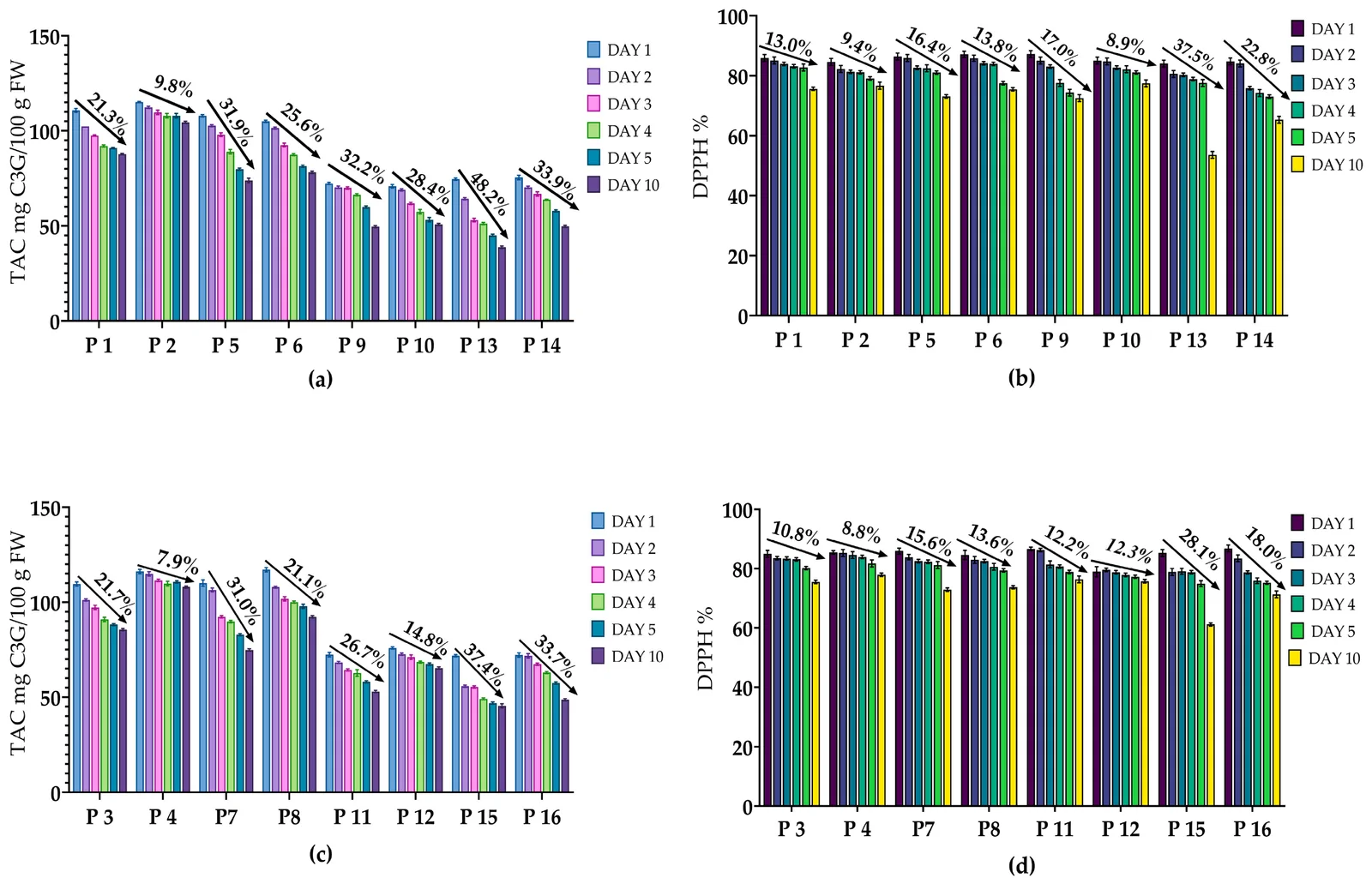
Changes in the anthocyanin content and antioxidant activity (DPPH) of red cabbage extract under aerobic and anaerobic conditions during storage. (**a**) anthocyanin content under aerobic conditions; (**b**) antioxidant activity (DPPH) under aerobic conditions; (**c**) anthocyanin content under anaerobic conditions; and (**d**) antioxidant activity (DPPH) under anaerobic conditions.

In the aerobic environment, the lowest TAC degradation (Figure 7a) was recorded for P2, whose anthocyanin content decreased from 115.470 mg CGE/100 g FW on day 1, to 107.321 mg CGE/100 g FW on day 5, and reached 104.161 mg CGE/100 g FW on day 10, corresponding to a low degradation rate (kTAC = 0.0112 day^−1^) and a half-life of 60.5 days. The greatest degradation was observed in sample P13, where TAC decreased from 74.273 mg CGE/100 g FW to 44.663 mg CGE/100 g FW on day 5, and on day 10 to 38.482 mg CGE/100 g FW, exhibiting the highest degradation rate (kTAC = 0.073 day^−1^) and the shortest half-life (9.5 days).

A similar trend was also observed for antioxidant activity (Figure 7b). The greatest stability was recorded for sample P10, where the DPPH value decreased from 85.718% on day 1 to 80.301% on day 5 and to 78.133% on day 10, corresponding to a kDPPH of 0.0103 day^−1^ and a half-life (t½) of 67.2 days. Sample P13 showed the most pronounced decrease in antioxidant activity, with the DPPH value decreasing from 84.718% to 78.234% on day 5 and to 52.905% on day 10, with a kDPPH of 0.052 day^−1^ and a t½ of 13.2 days.

In the anaerobic environment, the greatest TAC stability (Figure 7c) was observed in sample P4, whose anthocyanin content decreased from 116.859 mg CGE/100 g FW on day 1 to 110.353 mg CGE/100 g FW on day 5 and to 107.653 mg CGE/100 g FW on day 10, corresponding to a kTAC of 0.009 day^−1^ and a t½ of 76.0 days. In contrast, sample P15 showed the greatest degradation, with a kTAC of 0.052 day^−1^ and a t½ of 13.3 days.

In the case of DPPH antioxidant activity (Figure 7d), the smallest decrease was observed in sample P4, where the DPPH value decreased from 85.124% on day 1 to 81.070% on day 5 and to 77.626% on day 10, characterized by the lowest kDPPH (0.010 day^−1^) and the longest half-life (t½) (67.7 days). In contrast, sample P15 exhibited the most pronounced decline in antioxidant activity, with a kDPPH of 0.036 day^−1^ and a t½ of 18.9 days.

The presence of oxygen did not significantly affect any of the parameters (TAC: *p* = 0.373; DPPH: *p* = 0.641) and did not alter the rate of degradation during storage (day × oxygen: *p* = 0.314 and *p* = 0.242, respectively).

#### 2.4.3. The Effect of Light on Anthocyanin Content and Antioxidant Activity (DPPH)

Light has a dual effect on anthocyanins; it is essential for their biosynthesis in plant tissues, yet it has been shown that, after extraction, exposure to light accelerates their degradation [33,34]. The mechanism of photolytic degradation involves the breakdown of cyanidin-3-O-glucoside through the formation of intermediate products: after the release of the carbohydrate moiety (glucose at the C3 position) and the opening of the heterocyclic ring, the corresponding chalcone is formed. Subsequently, the chalcone degrades into low-molecular-weight compounds, namely 2,4,6-trihydroxybenzaldehyde (phloroglucinaldehyde), derived from ring A, and protocatechuic acid (3,4-dihydroxybenzoic acid), derived from ring B [20].

**Figure 8:**
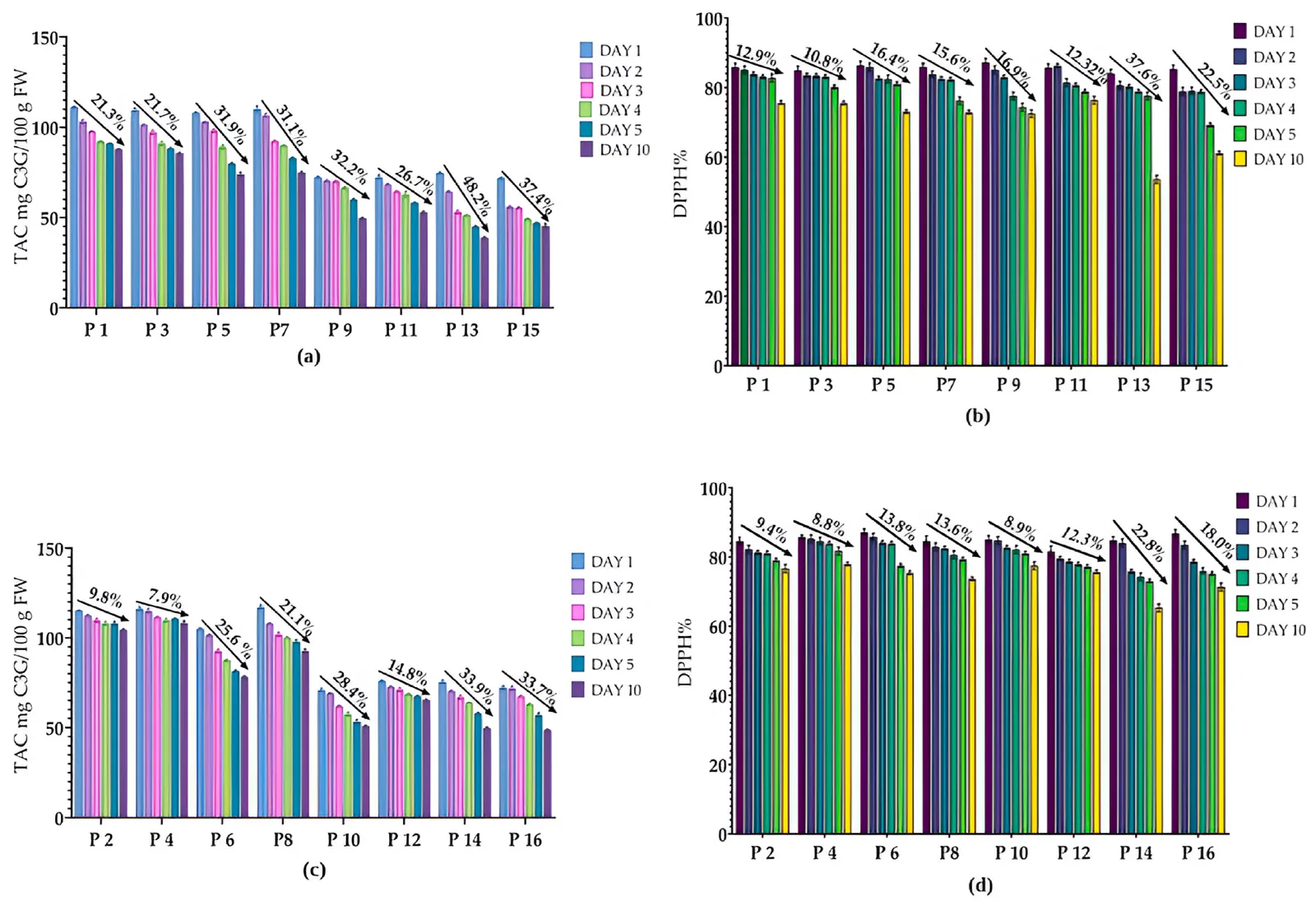
Changes in the anthocyanin content and antioxidant activity (DPPH) of red cabbage extract under light and dark conditions during storage. (**a**) anthocyanin content under light conditions; (**b**) antioxidant activity (DPPH) under light conditions; (**c**) anthocyanin content under dark conditions; (**d**) antioxidant activity (DPPH) under dark conditions.

Under light exposure, anthocyanin degradation was significant over the 10-day storage period (Figure 8a). The lowest TAC degradation was recorded in sample P1, whose anthocyanin content decreased from 111.370 mg CGE/100 g FW on day 1 to 90.818 mg CGE/100 g FW on day 5 and reached 87.632 mg CGE/100 g FW on day 10, corresponding to a kTAC of 0.027 day^−1^ and a t½ of 26.0 days. The greatest degradation was observed in sample P13, where the TAC decreased from 74.273 mg CGE/100 g FW to 44.663 mg CGE/100 g FW on day 5, and on day 10 it reached a value of 38.482 mg CGE/100 g FW, corresponding to a kTAC of 0.073 day^−1^ and a t½ of 9.5 days.

A similar trend was observed for antioxidant activity (Figure 8b). The highest DPPH stability was recorded in sample P3, where the value decreased from 84.313% on day 1 to 79.847% on day 5 and reached 75.194% on day 10, characterized by a k of 0.013 day^−1^ and a t½ of 54.5 days. Sample P15 showed the most pronounced decrease in antioxidant activity, with the DPPH value decreasing from 84.617% to 68.913% on day 5 and to 60.807% on day 10, with a k of 0.037 day^−1^ and a t½ of 18.9 days.

Storage in the dark resulted in better preservation of anthocyanins compared to exposure to light (*p* = 0.031), (Figure 8c). The highest TAC stability was observed in sample P4, whose anthocyanin content decreased from 116.859 mg CGE/100 g FW on day 1, to 110.353 mg CGE/100 g FW on day 5, and reached 107.653 mg CGE/100 g FW on day 10, corresponding to a kTAC of 0.009 day^−1^ and a t½ of 76.0 days. The greatest degradation was observed in sample P14, where the TAC decreased from 74.798 mg CGE/100 g FW to 58. 212 mg CGE/100 g FW on day 5 and decreased to 49.402 mg CGE/100 g FW on day 10, exhibiting a kTAC of 0.046 day^−1^ and a t½ of 15.0 days.

In the case of antioxidant activity (Figure 8d), the DPPH values followed a similar trend, with no statistically significant difference between the samples exposed to light and those stored in the dark (*p* = 0.741). The smallest loss was observed in sample P4, where the DPPH value decreased from 85.124% on day 1 to 81.070% on day 5, and on day 10 it reached 77.626%, characterized by the lowest kDPPH (0.010 day^−1^) and the longest t½ (67.7 days). In contrast, sample P14 exhibited the most pronounced decrease in antioxidant activity, with the DPPH value decreasing from 85.427% on day 1 to 73.371% on day 5 and to 65.975% on day 10, with a kDPPH of 0.029 day^−1^ and a t½ of 24.1 days.

#### 2.4.4. The Effect of Temperature on Anthocyanin Content and Antioxidant Activity (DPPH)

Temperature is the factor that has the strongest effect on the stability of anthocyanins in red cabbage; an increase in temperature reduces the stability of anthocyanins during storage [12].

**Figure 9:**
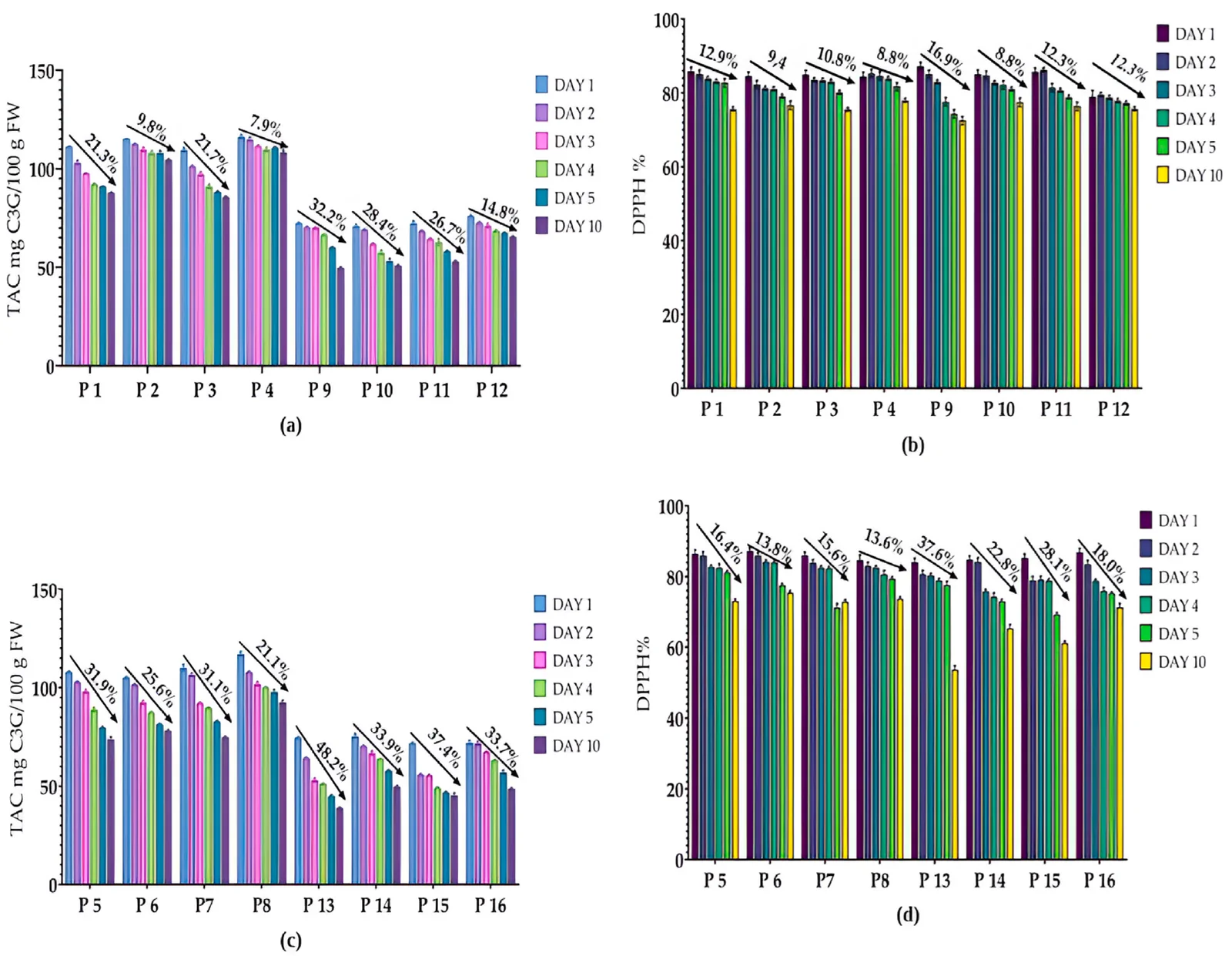
Changes in the anthocyanin content and antioxidant activity (DPPH) of red cabbage extract stored at 4 °C and 25 °C. (**a**) Anthocyanin content at 4 °C; (**b**) antioxidant activity (DPPH) at 4 °C; (**c**) anthocyanin content at 25 °C; (**d**) antioxidant activity (DPPH) at 25 °C.

At room temperature (Figure 9a), the highest TAC stability was observed in sample P4, whose anthocyanin content decreased from 116.859 mg CGE/100 g FW on day 1, to 110.353 mg CGE/100 g FW on day 5, and reached 107.653 mg CGE/100 g FW on day 10, corresponding to a degradation constant k = 0.009 days^−1^ and a half-life t^1/2^ = 76.0 days. The greatest degradation of TAC was observed in sample P9, where TAC decreased from 72.678 mg CGE/100 g FW to 59.587 mg CGE/100 g FW on day 5 and to 49.249 mg CGE/100 g FW on day 10, with kTAC = 0.043 days^−1^ and t^1/2^ = 16.0 days.

A similar trend was also observed for antioxidant activity (Figure 9b). The highest DPPH stability was recorded for sample P4, where the value decreased from 85.124% on day 1 to 81.071% on day 5 and reached 77.626% on day 10 (kDPPH = 0.010 days^−1^, t^1/2^ = 67.7 days). Sample P7 showed the most pronounced decrease in antioxidant activity, with the DPPH value decreasing from 85.934% to 70.534% on day 5 and to 72.560% on day 10, with a slight recovery in the second half of the storage period (kDPPH = 0.017 day^−1^, t^1/2^ = 41.9 days).

At 25 °C, the degradation of anthocyanins was significantly faster compared to storage at 4 °C (day × temperature: *p* = 0.006) (Figure 9c). The highest TAC stability was observed in sample P8, whose anthocyanin content decreased from 116.462 mg CGE/100 g FW on day 1 to 97.179 mg CGE/100 g FW on day 5 and reached 91.940 mg CGE/100 g FW on day 10, corresponding to a degradation constant kTAC = 0.026 days^−1^ and a half-life t^1/2^ = 26.4 days. The greatest degradation was observed in sample P13, where TAC decreased from 74.273 mg CGE/100 g FW to 44.663 mg CGE/100 g FW on day 5 and to 38.482 mg CGE/100 g FW on day 10, with kTAC = 0.073 days^−1^ and t^1/2^ = 9.5 days.

In terms of DPPH antioxidant activity (Figure 9d), the smallest decrease was observed in sample P4, where the DPPH value dropped from 85.124% on day 1 to 81.070% on day 5, and on day 10 it reached 77.626%, suggesting a slight stabilization in the second half of the storage period (kDPPH = 0.010 days^−1^, t^1/2^ = 67.7 days). In contrast, sample P13 showed the most pronounced decrease in antioxidant activity, with the DPPH value decreasing from 84.718% on day 1 to 78.234% on day 5 and to 52.905% on day 10 (kDPPH = 0.052 day^−1^, t^1/2^ = 13.2 days).

### 2.5. Analysis of the Microbiological Load in Red Cabbage Extract

**Figure 10:**
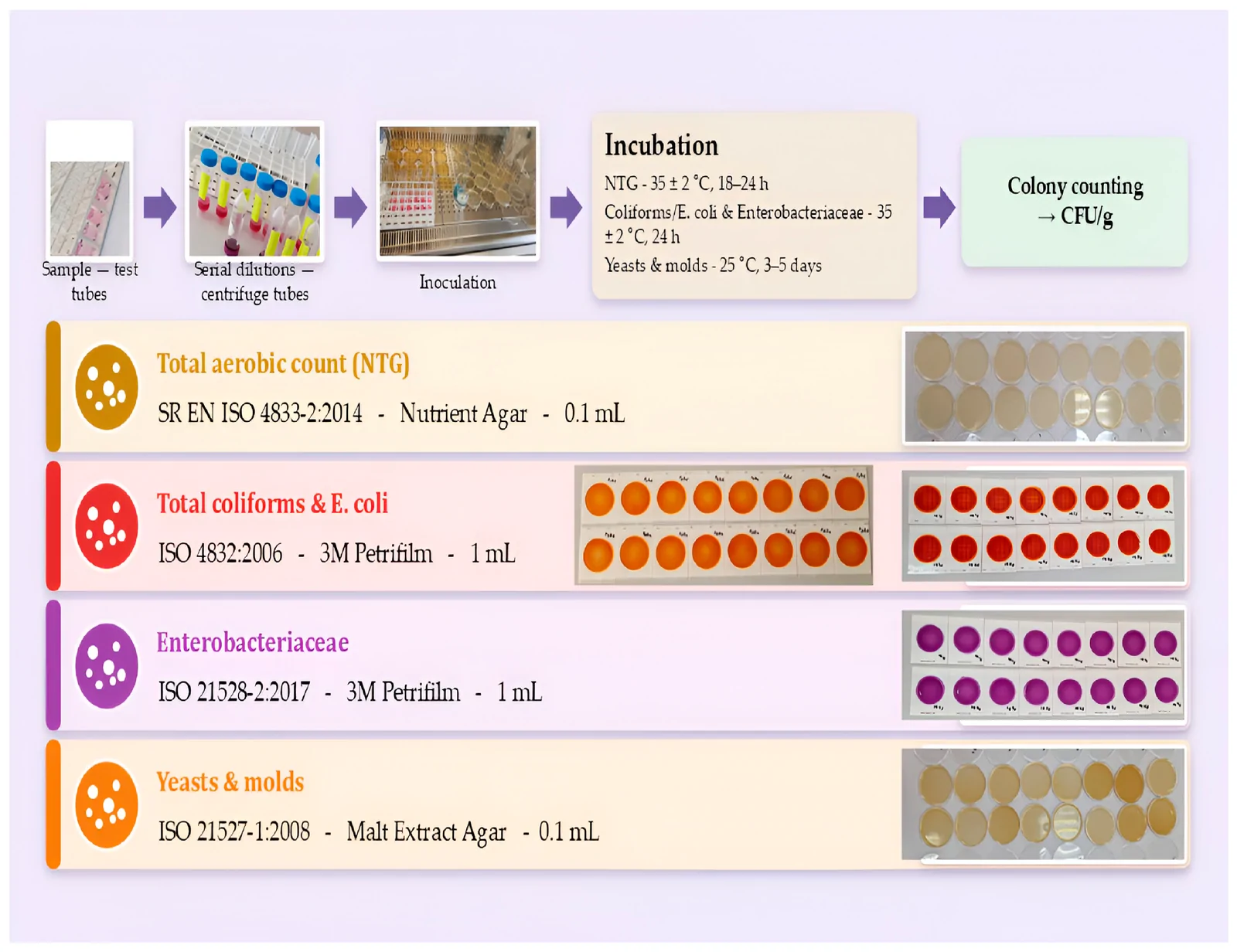
Microbiological analysis of the cabbage extract.

Following the analyses performed, the total aerobic mesophilic count (TAC) was determined in accordance with SR EN ISO 4833-2:2014 [35], using inoculation on Nutrient Agar; the total coliform and E. coli count was determined according to ISO 4832:2006 [36], using 3M Petrifilm plates, the Enterobacteriaceae count was determined according to ISO 21528-2:2017 [37], also using the 3M Petrifilm method and the yeast and mold count was determined according to ISO 21527-1:2008 [38], using inoculation on Malt Extract Agar (Figure 10). The results obtained were expressed in colony-forming units per gram of product (CFU/g), based on the counting of colonies developed after the incubation period specific to each parameter.

Microbiological characterization of red cabbage extracts indicated the absence of Enterobacteriaceae (REB), Escherichia coli (REC), and coliform bacteria (RCC) in all samples analyzed throughout the entire storage period (Table 4).

The values for total aerobic mesophilic bacteria (NTG) and yeasts and molds (YM) varied depending on storage conditions and the extraction solvent. In general, extracts obtained with 50% ethanol exhibited superior microbiological stability compared to aqueous extracts, maintaining lower TAG and YM values throughout the storage period. For example, sample P3 (ethanolic extract, stored at 4 ± 0.5 °C, in the dark, and under aerobic conditions) showed no microbial growth until day 5, and after 10 days, recorded only 200 CFU/g for NTG and 100 CFU/g for yeasts and molds. In contrast, the highest values were recorded for the aqueous extracts stored at 25 ± 0.5 °C, with sample P16 showing the highest NTG value (900 CFU/g), and sample P15 the highest yeast and mold count (1.300 CFU/g) after 10 days of storage.

Among the factors investigated, storage temperature had the most pronounced influence on microbial growth, with samples stored at 25 ± 0.5 °C generally exhibiting higher NTG and YM values compared to those stored at 4 ± 0.5 °C, while the effects of light and oxygenation were less evident.

## 3. Discussion

### 3.1. Physicochemical Parameters

The compositional characterization of the raw material was performed to evaluate the factors that may influence extraction efficiency and the stability of the extracts. Water content affects the solid–solvent ratio and the effective ethanol concentration, while water activity and pH can influence the structural stability of anthocyanins. At the same time, carbohydrates and phenolic compounds may contribute to their stabilization through copigmentation mechanisms. Determining these parameters allowed us to correlate the composition of the raw material with the behavior of the extracts under the stability conditions investigated.

The physicochemical values determined for fresh red cabbage in this study were generally consistent with those reported in the literature. The study conducted by Mejías et al. (2024), which evaluated the composition of fresh and dehydrated red cabbage, found a water content of 92.59% and a water activity of 0.993 for the fresh sample—values very close to those obtained in this study (91.72% and 0.98, respectively) [39].

In the study conducted by Stoica et al. (2023), the physicochemical analysis of red cabbage revealed a carbohydrate content ranging from 0.7 to 5.3%, fiber content ranging from 1.0 to 3.6%, protein content ranging from 0.8 to 2.0%, and mineral content ranging from 0.3 to 0.7% [29].

Similar results were also reported by Nwandu et al. (2024), who analyzed the composition of red and white cabbage, both in fermented and unfermented states, and found the following values for unfermented red cabbage: 24.05% moisture, 1.71% ash, 3.46% crude fiber, 1.05% fat, 19.85% protein, and 49.88% carbohydrates [40]. Hossain et al. (2026) characterized dried red cabbage powder and found it to contain 7.03% moisture, 14.67% ash, 2.89% crude fiber, 1.98% lipids, 22.68% protein, and 51.88% carbohydrates [41]. Although the values were reported on a dry matter basis, the observed nutritional profile was similar to that determined in the present study.

The differences observed between the results reported in the literature and those obtained in this study were minor and can be attributed to variety-specific characteristics, the degree of maturity, climatic growing conditions, and the analytical methodologies used [39]. However, these variations do not change the overall trend, confirming that red cabbage is characterized by a high water and carbohydrate content, a moderate level of protein and fiber, and a low lipid content.

### 3.2. Color Variation Depending on the pH of the Environment

The variation in color parameters as a function of pH observed in this study reflects the structural transformations of anthocyanins: from the flavyl cation (red) in an acidic environment to the quinoid bases (violet–blue) and to the ionized forms (blue–green) in an alkaline environment [42]. These transformations explain the progressive decrease in the a* parameter and the color shift observed experimentally, and the result is supported by several studies on anthocyanins in red cabbage.

An analysis of pH-dependent color variation in red cabbage extract was conducted by Abedi-Firoozjah et al. (2022) [43]. The authors reported a gradual change in color over the pH range of 1–11, from red hues in a strongly acidic environment (pH 1–2) to pink and purple in the weakly acidic range (pH 3–6), followed by blue tones at pH 7–8 and green in an alkaline environment (pH 9–11). A similar finding is presented in the review by Ghareaghajlou et al. (2021) on anthocyanins in red cabbage, which show a color change from reddish-purple to bluish-green over the pH range of 2–9 [12]. These results are consistent with those obtained in the present study, where the a* parameter was positive in an acidic environment and had negative values in an alkaline environment, indicating a shift in color toward bluish-green hues, while the negative values of the b* parameter highlighted the predominance of the blue component across the entire pH range analyzed.

Along the same lines, Nadi et al. (2023) evaluated the color response of films containing red cabbage extract and obtained red colors in an acidic medium, blue colors in a neutral medium, and green colors in an alkaline medium, while also reporting that the inclusion of red cabbage extract increased the total color difference (ΔE) of the films [2]. Echegaray et al. (2022) showed that exposure to high temperatures, light, and oxygen accelerates the degradation of anthocyanins, leading to color changes and reduced pigment stability [26]. This observation is consistent with the results obtained in the present study, where samples stored at 25 °C in the presence of light and oxygen exhibited the greatest changes in color parameters and stability indices.

### 3.3. Stability of Red Cabbage Extract Under Environmental Conditions

The results showed that the stability of anthocyanins in red cabbage extracts was influenced by both the type of extraction solvent and storage conditions. The 50% ethanol extracts showed higher initial values for total anthocyanin content (TAC) compared to the aqueous extracts, and this difference persisted throughout the storage period. These results indicate that 50% ethanol is more effective than water at recovering anthocyanin compounds from red cabbage and support its selection as a more promising solvent for obtaining an extract intended for the further development of a colorimetric indicator. Regarding antioxidant activity, both types of extracts showed a progressive decrease in DPPH values during storage, indicating a decline in antioxidant capacity over time. Compared to the aqueous extracts, the ethanol extracts generally maintained more favorable antioxidant activity values; this finding, combined with the higher TAC values, supports the potential of 50% ethanol extraction for future applications. These observations are consistent with the literature, which highlights the protective role of ethanol. Patras (2019) demonstrated that the hydroethanolic extract of red cabbage (48% ethanol) is significantly more stable than the aqueous extract, as ethanol reduces water activity (Aw), thereby limiting the hydration reactions of flavylium cations and the conversion of quinonoid bases into colorless compounds [31]. This finding is also supported by the study conducted by Oancea (2021), which highlighted the role of water activity in anthocyanin degradation processes and the importance of reducing it to preserve pigment stability [21]. Stoica et al. (2023) confirmed that a 50% ethanol–water mixture acidified with apple cider vinegar yielded extracts with good stability for food applications, with anthocyanin retention remaining high throughout storage [29].

#### 3.3.1. Influence of Oxygen

The highest stability was observed under anaerobic conditions, in the dark, and at a temperature of 4 °C, where lower losses in total anthocyanin content (TAC) and antioxidant activity, as determined by the DPPH method, were recorded compared to the other storage conditions. These results are consistent with the scientific literature, which highlights that the stability of anthocyanins is strongly influenced by environmental factors, particularly temperature, the presence of oxygen, and exposure to light, which are considered the main factors responsible for structural degradation and color loss [12,20,33,44]. The authors emphasize that limiting exposure to these factors is essential for preserving the antioxidant properties of anthocyanin-rich extracts.

#### 3.3.2. Influence of Temperature

Temperature was an important factor in degradation in this study, with extracts stored at 25 °C showing greater losses of TAC and DPPH compared to those stored at 4 °C. The more pronounced degradation observed at high temperatures can be explained by the acceleration of the chemical reactions involved in the instability of these compounds. In the case of anthocyanins, rising temperatures promote the hydrolysis of glycosidic bonds, leading to the formation of aglycones, which are subsequently more susceptible to degradation. Thermal processes also cause the opening of the pyran ring, resulting in the formation of chalcone, which breaks down into phenolic acids and aldehydes [45]. Concurrently, oxidation and condensation reactions occur, leading to the formation of brown polymeric pigments—a process supported in this study by the significant increase in polymeric color and the browning index during storage, accompanied by a decrease in color density and total anthocyanin content. The results obtained confirm the trends reported in the literature regarding the effect of temperature on the stability of antioxidant activity and anthocyanins in red cabbage. The study conducted by Sendri et al. (2023), which analyzed the stability of red cabbage extracts over 120 days at 4 °C and 25 °C, showed a decrease in anthocyanin retention as temperature and storage duration increased, with the lowest loss recorded at 4 °C [46]. The final anthocyanin content was 74.52 ± 0.37 at 4 °C, compared to 69.58 ± 0.54 at 25 °C, demonstrating that lower temperatures help maintain anthocyanin stability during storage. Similarly, De Marchi et al. (2024) studied the thermal degradation kinetics of anthocyanins in an aqueous red cabbage extract at 40 °C for 30 days and demonstrated that the degradation follows first-order kinetics, with an overall half-life of 16.4–18.4 days, ranging from 12.6 to 35.1 days depending on the molecular structure of each anthocyanin [47]. Kurek et al. (2024), in a study conducted on red cabbage extracts stored at −18 °C, 4 °C and 25 °C for 35 days, determined the degradation kinetics of anthocyanins by calculating the degradation constant (k) and the half-life (t^1/2^) [30]. It was found that the highest degradation rates were recorded at 25 °C, with half-lives of approximately 46 days, while storage at 4 °C resulted in significantly higher stability, with t^1/2^ reaching 166.7 days. At freezing temperature (−18 °C), degradation was also slower compared to room temperature, with a t^1/2^ of 131.4 days. In a study conducted on red cabbage extracts stored at −18 °C, 4 °C, and 25 °C for 35 days, it was found that the highest degradation rates were recorded at 25 °C, while storage at 4 °C resulted in significantly higher stability. The authors explain these differences by the fact that at refrigeration temperatures (4–5 °C), enzymatic activities are slowed down, which reduces degradation compared to room temperature. The downward trend in total anthocyanin content (TAC) and antioxidant activity, as determined by the DPPH method, with increasing temperature was also highlighted by Vega-Gálvez et al. (2023), who demonstrated the sensitivity of these compounds to thermal degradation and the decline in antioxidant capacity at high temperatures [10].

#### 3.3.3. Influence of Light

In the experiment, the most stable samples were those stored in the dark, a finding that is consistent with the literature. The study by Ghareaghajlou et al. (2021) showed that light is one of the most important factors that reduce the stability of anthocyanins and the antioxidant activity of red cabbage extracts [12]. Similarly, Enaru et al. (2021) confirmed that light intensity and exposure duration directly influence the degradation rate, with more pronounced effects under UV radiation than under visible light [44]. These results are supported by data from the literature; Sendri et al. (2023) reported that the retention of cyanidin-3,5-O-diglucoside in unencapsulated samples exposed to sunlight was only 1.76%, and that of cyanidin-3-O-glucoside was 0.17%, compared to significantly higher values in samples stored in the dark [46]. Furthermore, Ionescu et al. (2025), in their study of blueberry extracts in an ethanol/water solution, stored under four experimental conditions for 10 days, found that the most pronounced degradation of anthocyanins occurred under conditions of exposure to sunlight, with the degree of degradation (CR) reaching 97.4% on day 10 [32].

### 3.4. Analysis of Colorimetric Indices

The changes observed for the browning index (BI), polymeric color (PC), color density (CD), and degradation index (DI) are consistent with the trends reported in the literature for anthocyanin-rich extracts. Recent studies show that storage at high temperatures accelerates the degradation of anthocyanins, leading to an increase in the browning index, polymeric color, and degradation index, accompanied by a decrease in color density.

In their study, Stoica et al. (2023) characterized anthocyanin extracts from red cabbage byproducts obtained using ethanol/water and apple cider vinegar, determining the color density and degradation index and evaluating stability during storage [29]. The authors found that the aqueous extract had a degradation index of approximately 1.05 and a low color density (1.05), while the acidified ethanolic extract had a significantly lower DI (0.21–0.27) and the highest color density (4.12). This difference confirms the progressive reduction in stability in the aqueous extracts and is consistent with the more pronounced increase in the degradation index and the more marked decrease in color density observed in the aqueous samples in the present study compared to the ethanolic extracts.

A similar study, conducted by Patras (2019), evaluated a hydroethanolic extract (48% ethanol) of red cabbage over a 20-week storage period at 4 °C in the dark by determining color density, polymeric color, the browning index, and the degradation index [31]. The ethanolic extracts of red cabbage exhibited color density values ranging from 2.30 to 3.07 and polymeric color values ranging from 0.24 to 0.32. These results are comparable to those obtained in the present study, where the ethanolic extracts exhibited lower polymeric color (PC) values and a slight decrease in color density (CD) compared to the aqueous extracts, indicating better color stability.

In addition, Kurek et al. (2024) investigated red cabbage extracts stored at 4 °C, 25 °C and −18 °C [30]. The results showed an increase in the browning index and polymeric color as storage temperature rose, while refrigeration more effectively preserved the anthocyanin compounds. These observations are similar to those obtained in the present study, where samples stored at 25 °C showed the greatest increases in BI, PC, and DI.

## 4. Materials and Methods

### 4.1. Raw Materials

Red cabbage (*Brassica oleracea* L. var. *capitata* f. *rubra*) was purchased from a local supermarket (Kaufland, Romania) in Suceava, Romania (47.65° N, 26.26° E).

### 4.2. Sample Preparation

The fresh red cabbage samples were mechanically homogenized until a uniform mass was obtained. Anthocyanin extraction was performed using distilled water and a 50% (*v*/*v*) ethanol hydroalcoholic solution, at a solid-to-solvent ratio of 1:10 (*m*/*v*). The extracts were adjusted to pH 3 by adding 0.1 N HCl to stabilize the anthocyanins. A single extract batch was prepared for each solvent, and the extracts were distributed into 32 storage flasks: 16 for the physicochemical and antioxidant analyses and 16 for the microbiological analyses. Storage conditions followed a full factorial design: 2 solvents × 2 light conditions × 2 oxygen conditions × 2 temperatures, resulting in 16 experimental combinations (Table 5).

For the physicochemical and antioxidant analyses, one flask was used for each of the 16 experimental combinations. Each flask constituted an experimental unit and was maintained throughout the experiment, with samples withdrawn successively from the same flask on days 1, 2, 3, 4, 5 and 10 of storage. All determinations were performed in triplicate, and the mean of the three readings was used as the value of the experimental unit. The remaining 16 flasks were used exclusively for the microbiological analyses, following the same sampling schedule.

For the evaluation of pH-dependent color variation, both extracts were examined at pH values ranging from 1 to 10. For each pH value, two samples were prepared, one for the ethanolic and one for the aqueous extract. The same samples were monitored throughout the storage period in order to follow the evolution of the color parameters over time, with instrumental determinations performed in triplicate for each sample.

### 4.3. Maceration Extraction (MAC)

MAC is a conventional method that involves immersing plant material in a solvent. The method was used to extract anthocyanins from fresh red cabbage samples. Two solvents were used for the extraction: distilled water and 50% (*v*/*v*) ethanol, each applied in the same solid-to-solvent ratio of 1:10 (*m*/*v*). The sample-solvent mixture was subsequently acidified to pH 3 by adding 0.1 N HCl to stabilize the anthocyanins. Maceration was carried out at room temperature (25 °C) for 24 h in the absence of light. After extraction, the samples were centrifuged at 5000 rpm for 20 min at 25 °C. Filtration was performed in two successive stages using Whatman No. 1 filter paper, followed by filtration through Millipore syringe filters (Merck, Darmstadt, Germany) with a pore size of 0.45 μm [12,48].

### 4.4. Physicochemical Characterization of Red Cabbage

#### 4.4.1. Moisture Content

The moisture content was determined according to AOAC Official Method 925.10 [49]. Exactly 5 g of the sample was weighed and dried in a forced-air oven (Memmert UFB500, Memmert GmbH + Co. KG, Schwabach, Germany) at 103 ± 2 °C until a constant mass was achieved. After drying in the oven, the sample was transferred to a desiccator and allowed to reach room temperature before weighing [50,51,52,53,54,55]. Formula (1) was used to determine the moisture content:

(1) $$Moisture\;(\%)=\frac{W_{1}-W_{2}}{W_{1}}$$

where W_1_ is the mass (g) of the sample before drying, and W_2_ is the mass (g) of the sample after drying.

#### 4.4.2. Ash Content

The ash content was determined according to Official Method 940.26 of AOAC International [56] by calcining 5 g of the sample at 550–600 °C in a Nabertherm L9 C6 calcination furnace (Nabertherm, Lilienthal, Germany) until a constant mass was obtained [57,58,59].

(2) $$Ash\;(\%)=\frac{Initial\;sample\;mass\;(g)}{Mass\;of\;the\;residue\;after\;calcination\;(g)}\times 100$$

#### 4.4.3. Fiber Content

Crude fiber was determined according to the standardized method (ISO 5498:1981) [60] using a FIBERTHERM FT12 analyzer (C. Gerhardt, Königswinter, Germany). Exactly 2 g of each sample was weighed and placed in FiberBag. The samples were then subjected to successive digestion in acidic and alkaline media. The residues were dried in an oven at 105 °C to constant weight, then calcined at 550 °C [61,62,63,64]. The crude fiber content was calculated according to Equation (3):

(3) $$Crude\;fiber\;(\%)=\frac{\left(W_{2}-W_{3}\right)-W_{4}}{W_{1}}\times 100$$

where W_1_ is the mass of the analyzed sample (g), W_2_ is the mass of the FiberBag bag with the dry residue after acid and alkaline digestion (g), W_3_ is the mass of the empty FiberBag bag, and W_4_ is the mass of the ash obtained after calcination of the residue.

#### 4.4.4. Protein Content

The crude protein content of the red cabbage samples was determined using the Kjeldahl method, which involves digestion, distillation, and titration. The protein content was calculated based on the nitrogen content, using a conversion factor of 5.65 [36,65]. The calculation was performed according to formula (4):

(4) $$Protein\;(\%)=\frac{\left(V\;sample-V\;blank\right)\times Z\times C\times f\times MN}{m\times 1000}\times 5.65$$

where V sample is the volume of HCl used to titrate the sample (mL), V blank is the volume of HCl used to titrate the control sample (mL), z is the stoichiometric factor (1 for HCl), C-concentration of HCl (mol/L), f is the correction factor for the HCl solution (1), MN is the mass of nitrogen (14.007 g/mol), m is the mass of sample (g), 1000 is the conversion from mL to L.

#### 4.4.5. Lipid Content

The total lipid content was determined using the Soxhlet method, in accordance with AOAC Official Method 920.39. The fresh sample was subjected to continuous extraction with petroleum ether for 6–8 h. After removing the solvent by evaporation, the lipid residue was dried to constant weight and weighed gravimetrically. The lipid content was expressed as g of lipids per 100 g of fresh weight (g/100 g FW) [40,41].

(5) $$Lipid\;(\%)=\frac{\left(m_{2}-m_{1}\right)}{m}\times 100$$

where m_1_ is the mass of the empty extraction flask (g), m_2_ is the mass of the extraction flask after solvent evaporation (g), and m is the mass of the analyzed sample (g).

#### 4.4.6. Carbohydrate Content

The carbohydrate content was determined using the difference method, by subtracting the sum of the other macronutrients from the total weight of the product. The calculation was performed using formula (6) [41].

Carbohydrates (%) = 100 − (Protein (%) + Fat (%) + Moisture (%) + Ash (%) + Fiber (%))(6)

#### 4.4.7. Water Activity (aw)

Water activity (aw) was determined at 25 °C in triplicate using an AquaLab 4TE device (Meter Group, Pullman, Washington, DC, USA) [39].

#### 4.4.8. pH

The pH was determined using a Mettler Toledo pH meter (Seven Compact, Heusenstamm, Germany).

### 4.5. Total Anthocyanin Content (TAC)

The anthocyanin content of the red cabbage extract was determined using the differential pH method. Analyses were performed on days 1, 2, 3, 4, 5, and 10 to evaluate changes in anthocyanin content over time. Total anthocyanin content was measured using the differential pH spectrophotometric method. The extract was adjusted with a potassium chloride (KCl) buffer solution (pH 1, 0.025 mol L^−1^) and a sodium acetate (CH_3_COOH) buffer solution (pH 4.5, 0.5 mol L^−1^). The buffer solution was incubated for 15 min in the dark. Measurements were performed using a Shimadzu 300 UV-VIS-NIR spectrophotometer (Tokyo, Japan) at wavelengths of 510 nm and 700 nm. Total anthocyanin content (TAC) was calculated using Equations (7) and (8). It was expressed as cyanidin-3-glucoside equivalents (mg C3G/100 g FW) [46,66,67,68].

A = (A_520_ − A_700_)_pH 1_ − (A_520_ − A_700_)_pH 4.5_ (7)

(8) $$TAC=\frac{A\times mw\times DF\times V\;(mL)}{\varepsilon \times m\left(g\right)\times L}$$

where A is (A_520nm_ − A_700nm_) at pH 1.0 and (A_520nm_ − A_700nm_) at pH 4.5; mw is the molecular weight of cyanidin-3-glucoside (C3G) (449.2 g/mol); DF is the sample dilution factor; V is the sample volume (mL); ε is the molar extinction coefficient of C3G (26,900 L·mol^−1^·cm^−1^); m is the sample mass (g); and L is the cell length (1 cm).

### 4.6. Antioxidant Activity (DPPH)

The antioxidant activity of the extract was determined using the DPPH (2,2-diphenyl-1-picrylhydrazyl) free radical scavenging assay. Analyses were performed on days 1, 2, 3, 4, 5 and 10 to monitor changes in antioxidant capacity over time. An undiluted 0.1 mL sample of the extract was mixed with 3.9 mL of DPPH solution and incubated for 30 min in the dark. After 30 min, the absorbance was measured at 517 nm using a spectrophotometer (Shimadzu 300 UV-VIS-NIR, Tokyo, Japan) [10].

(9) $$Antioxidant\;activity\left(\%\right)=\frac{Ablank-Asample}{Asample}\times 100$$

### 4.7. The Effect of pH and Storage Time on Color Parameters

To evaluate the influence of pH on color behavior, the extracts were adjusted to pH values ranging from 1 to 10 by titration with 0.1 N HCl and 0.1 N NaOH solutions, using a Mettler Toledo Seven Compact pH meter (Heusenstamm, Germany). Absorption spectra were recorded in the 400–700 nm range using a UV–Vis-NIR spectrophotometer (Shimadzu 300, Tokyo, Japan), and color parameters in the CIELAB system were determined using a Konica Minolta CM-700 colorimeter (Konica Minolta, Tokyo, Japan). The L*, a*, and b* parameters were recorded, where L* expresses lightness (0 = black, 100 = white), a* represents the green (–) to red (+) chromatic coordinate, and b* represents the blue (–) to yellow (+) chromatic coordinate [39,40]. Based on the CIELAB coordinates, the chroma (C*), which reflects color intensity, and the hue angle (H°) were calculated using Equations (10) and (11). Three measurements were performed for each sample, and color changes were monitored on days 1, 2, 3, 4, 5 and 10. The color stability of the extracts at different pH values was evaluated by calculating the total color difference (ΔE), obtained by comparing the colorimetric parameters determined on day 1 with those recorded on subsequent days, according to Equation (12) [55,69].

(10) $$C=\sqrt{{a^{*}}^{2}+{b^{*}}^{2}}$$

(11) H=arctana∗b∗

(12) $$\;\Delta E=\sqrt{{\left(\Delta L^{*}\right)}^{2}+{\left(\Delta a^{*}\right)}^{2}+{\left(\Delta b^{*}\right)}^{2}}$$

where ΔL*, Δa*, and Δb* represent the differences in colorimetric parameters between the values determined on Day 1 and those obtained on Days 2, 3, 4, 5 and 10.

The ΔE values were classified into six levels based on the National Bureau of Standards (NBS) scale, calculated as NBS = ΔE × 0.92: trace (0–0.5), slight (0.5–1.5), noticeable (1.5–3.0), appreciable (3.0–6.0), high (6.0–12.0), and very high (>12.0) [70].

### 4.8. Degradation Kinetics of Anthocyanins and Antioxidant Activity

The kinetics of anthocyanin degradation and antioxidant activity during storage were evaluated using a first-order model. The degradation rate constant (k, days^−1^) and half-life (t_1/2_, days) were calculated based on Equations (13) and (14), parameters used to characterize the stability of bioactive compounds over time [32,71]:

(13) $$C_{t}=C_{0}\cdot e^{-kt}$$

(14) $$t_{1/2}=-\frac{ln(0.5)}{k}$$

where C_0_ and C_t_ represent the anthocyanin content at the initial time and at time t, respectively; k is the degradation rate constant (1/h); and t is the storage time.

### 4.9. Determination of Chromatic Parameters (CD, PC, BI, DI)

Color density (CD), polymeric color (PC), browning index (BI), and degradation index (DI) were determined using the metabisulfite decolorization method [30]. For the control solution (W), 0.5 mL of extract was diluted with distilled water to a final volume of 10 mL. For the treated solution (B), 0.5 mL of extract was mixed with 1 mL of 20% sodium metabisulfite solution and diluted with distilled water to a final volume of 10 mL [29,72]. The samples were homogenized and allowed to stand at room temperature for 15–20 min. Absorbances were measured at 420 nm, 520 nm, and 700 nm, using distilled water as a control. All determinations were performed in triplicate (*n* = 3).

Color density (CD) was calculated using the values obtained for the water-treated sample (W), according to Equation (15) [29,31]:

(15) $$CD=[(A_{420}W-A_{700}W)+(A_{520}W-A_{700}W)]\times DF$$

The polymeric color (PC) was determined from the absorbances of the sample treated with metabisulfite (B) [31]:

PC = [(A_420_B − A_700_B) + (A_520_B − A_700_B)] × DF (16)

The browning index (BI) was calculated as the ratio of the absorbance at 420 nm to that at 700 nm (sample B) [31]:

BI = (A_420_B − A_700_B) × DF (17)

The degradation index (DI) was calculated according to Equation (18) [29,31]:

DI = (A_420_W − A_700_W)/(A_520_W − A_700_W) (18)

where A_420_W, A_520_W, and A_700_W represent the absorbance values measured at 420 nm, 520 nm, and 700 nm, respectively, for the sample treated with water (W); A_420_B, A_520_B, and A_700_B represent the absorbance values measured at 420 nm, 520 nm, and 700 nm, respectively, for the sample treated with metabisulfite (B); and DF represents the dilution factor.

### 4.10. Study of the Stability of Red Cabbage Extract

#### 4.10.1. Light Stability

The light stability of anthocyanins was evaluated by storing the samples under continuous exposure to ambient light and in total darkness. The samples were analyzed on days 1, 2, 3, 4, 5, and 10, in triplicate. The light stability of the extract was evaluated by monitoring changes in total anthocyanin content, antioxidant activity, color density (CD), browning index (BI), degradation index (DI), and polymeric color (PC) [73].

#### 4.10.2. Temperature Stability

The effect of storage temperature on anthocyanin stability was investigated by storing the extract at 4 °C (refrigeration) and at 25 °C (room temperature). For each sample, measurements were performed in triplicate and included total anthocyanin content (TAC), antioxidant activity (DPPH), and color parameters (CD, BI, DI, and PC).

#### 4.10.3. Oxidative Stability

The oxidative stability of anthocyanins was evaluated by comparing samples stored in the presence and absence of oxygen. At each time point, the main stability parameters were analyzed: total anthocyanin content (TAC), antioxidant activity, color density (CD), browning index (BI), degradation index (DI), and polymeric color (PC) [44].

### 4.11. Microbiological Analysis

The microbiological load of the cabbage extract was determined after performing successive decimal dilutions down to 10^−5^. The total number of coliforms and Escherichia coli was determined in accordance with ISO 4832/2006 [36], and ISO 21528-2:2017 [37] was used for the determination of Enterobacteriaceae. The analyses were performed using the 3M Petrifilm system by inoculating 1 mL of each dilution and incubating at 35 ± 2 °C for 24 h. The total number of aerobic mesophilic microorganisms (NTG) was determined in accordance with SR EN ISO 4833-2:2014 [35] on Nutrient Agar by spreading 0.1 mL of each dilution onto the surface and incubating at 35 ± 2 °C for 18–24 h. Yeasts and molds were determined in accordance with ISO 21527-1:2008 [38] on Malt Extract Agar, using 0.1 mL of inoculum, followed by incubation at 25 °C for 3–5 days. After incubation, the colonies were counted, and the results were expressed as colony-forming units per gram of sample (CFU/g), with only plates showing between 30 and 300 colonies being considered [74,75,76,77].

### 4.12. Statistical Analysis

All instrumental measurements were performed in triplicate for each sample; the average of the three readings was used as the value for the experimental unit, and the results are expressed as the mean ± standard deviation. For the experiment on color as a function of pH, a one-way ANOVA was applied, followed by Tukey’s HSD post-hoc test (*p* < 0.05). For CD, PC, BI, DI, DPPH, and TAC, a multifactorial ANOVA with repeated measures was applied, including interactions between the experimental factors, with the Greenhouse–Geisser correction. Statistical analyses were performed using Jamovi version 2.7 (The Jamovi Project, 2025), and graphs were generated using GraphPad Prism version 11 (GraphPad Software, San Diego, CA, USA).

## 5. Conclusions

Monitoring food freshness is a key area of interest in the development of smart packaging systems, as it allows for the rapid and visual assessment of changes that occur during storage. In this context, the anthocyanins in red cabbage are natural pigments with potential for use as colorimetric indicators due to their sensitivity to pH changes associated with the spoilage processes of perishable foods.

The results showed a gradual decrease in total anthocyanin content (TAC) and antioxidant activity (DPPH) over the 10-day storage period, regardless of the experimental conditions applied. The stability of the extracts was influenced by the solvent used for extraction, the presence of oxygen, light, and temperature. The novelty of this study lies in the simultaneous evaluation of these factors using a full factorial design, which allows for the analysis of individual effects and interactions among storage conditions. In addition, the study compares the stability of aqueous and ethanolic extracts of red cabbage by monitoring bioactive compounds, antioxidant activity, chromatic and spectrophotometric parameters, and microbial load.

A comparison of the two extraction methods indicated that ethanol extraction was more efficient: the extracts obtained had a higher initial content of phenolic compounds and anthocyanins, as well as greater stability compared to the aqueous extracts, and storage under anaerobic conditions further contributed to the preservation of bioactive compounds.

The study of color variation as a function of pH highlighted the characteristic behavior of anthocyanins, with a clear and reproducible transition observed from shades of red in an acidic environment to blue, greenish, and yellow in an alkaline environment. Colorimetric parameters demonstrated that the color changes are pronounced enough to be visually observed, which is essential for the use of the extract as a colorimetric indicator. In addition, the ethanolic extract exhibited low values of total color difference (ΔE) during storage, demonstrating superior color stability and greater resistance to degradation.

The microbiological load varied depending on storage conditions and the solvent used, with NTG and YM values generally lower for ethanolic extracts compared to aqueous extracts. Furthermore, no Enterobacteriaceae, Escherichia coli, or coliform bacteria were detected in the samples analyzed during the storage period. This finding, together with the observed chemical and colorimetric stability, supports the possibility of using the extracts in practical monitoring applications.

Overall, the results demonstrate that red cabbage ethanol extract exhibits properties suitable for the development of a natural colorimetric indicator for monitoring food freshness. Among the factors analyzed, temperature had the greatest influence on degradation, followed by light, while the effect of oxygen was less significant. Its high sensitivity to pH changes, distinct color variations, and superior stability under refrigerated conditions (4 °C) highlight its potential for integration into smart packaging systems, contributing to improved food safety and reducing food waste. However, the results obtained under the experimental conditions of this study represent a preliminary step and do not yet demonstrate the extract’s performance as an indicator integrated into a real-world packaging system. A limitation of the study is that the extracts were evaluated exclusively under controlled laboratory conditions, without integrating them into an indicator matrix and without direct validation on food products. Furthermore, it remains to be determined to what extent the extract’s color changes directly reflect changes in food freshness and spoilage under real-world storage conditions. Therefore, future research should focus on translating the results into practical applications by integrating the optimized extract into an indicator matrix compatible with packaging materials and validating it directly on food products by correlating the color response with the physicochemical and microbiological parameters of freshness. At the same time, future studies will determine the extract’s antimicrobial activity through specific tests to directly assess its potential for use in applications aimed at maintaining food quality and preservation. Assessing the indicator’s stability, compatibility with packaging materials, and performance under various temperature and packaging atmosphere conditions will be an essential step in transferring the results from the laboratory to practical applications in the field of smart packaging.

## Figures and Tables

**Table 1:** Physicochemical Characterization of Red Cabbage (*Brassica oleracea*).

Parameters	Red Cabbage (*B. oleracea*)
Moisture (%)	91.72 ± 0.74
Ash (%)	0.56 ± 0.007
Lipid (g/100 g FW)	0.10 ± 0.01
Crude Fiber (g/100 g FW)	0.71 ± 0.02
Protein (g/100 g FW)	1.01 ± 0.02
Total Carbohydrates (difference)	5.90
pH	6.17 ± 0.18
Water activity (A_W_)	0.98 ± 0.002

Data are presented as mean ± standard deviation (*n* = 3), where FW refers to fresh weight.

**Table 2:** Colorimetric characterization (CIELAB) of ethanolic extracts at different pH values.

pH	Day	L*	a*	b*	C*	h	ΔE
1	1	2.07 ± 0.09 ^a^	2.96 ± 0.30 ^a^	−0.77 ± 0.05 ^b^	3.05 ± 0.30 ^a^	345.4 ± 1.0 ^a^	—
1	2	2.64 ± 0.19 ^a^	3.22 ± 0.15 ^a^	−0.78 ± 0.15 ^b^	3.30 ± 0.18 ^a^	346.4 ± 2.0 ^a^	0.64 ± 0.23 ^a^
1	3	2.46 ± 0.73 ^a^	3.54 ± 0.61 ^a^	−0.44 ± 0.08 ^a^	3.57 ± 0.60 ^a^	352.1 ± 1.9 ^a^	0.91 ± 0.75 ^a^
1	4	2.37 ± 0.05 ^a^	2.96 ± 0.05 ^a^	−1.11 ± 0.05 ^c^	3.17 ± 0.07 ^a^	339.1 ± 0.8 ^b^	0.46 ± 0.00 ^a^
1	5	2.47 ± 0.61 ^a^	3.26 ± 0.26 ^a^	−0.97 ± 0.08 ^b^	3.40 ± 0.26 ^a^	342.9 ± 1.4 ^b^	0.60 ± 0.59 ^a^
1	10	2.08 ± 0.13 ^a^	3.14 ± 0.09 ^a^	−1.07 ± 0.06 ^c^	3.31 ± 0.11 ^a^	340.5 ± 1.8 ^b^	0.37 ± 0.07 ^a^
2	1	1.80 ± 0.01 ^a^	2.86 ± 0.11 ^a^	−1.09 ± 0.02 ^b^	3.11 ± 0.13 ^a^	339.5 ± 0.6 ^a^	—
2	2	2.27 ± 0.07 ^a^	2.96 ± 0.12 ^a^	−1.05 ± 0.01 ^b^	3.16 ± 0.09 ^a^	340.6 ± 0.6 ^a^	0.50 ± 0.06 ^b^
2	3	1.72 ± 0.51 ^a^	2.83 ± 0.49 ^a^	−0.74 ± 0.05 ^a^	2.92 ± 0.46 ^a^	344.9 ± 3.1 ^a^	0.67 ± 0.16 ^a^
2	4	2.16 ± 0.11 ^a^	2.40 ± 0.05 ^b^	−1.27 ± 0.09 ^c^	2.72 ± 0.08 ^a^	332.2 ± 1.3 ^b^	0.62 ± 0.09 ^a^
2	5	2.13 ± 0.63 ^a^	2.17 ± 0.07 ^b^	−1.27 ± 0.09 ^c^	2.52 ± 0.10 ^b^	329.8 ± 1.1 ^b^	0.91 ± 0.29 ^a^
2	10	1.83 ± 0.05 ^a^	2.17 ± 0.20 ^b^	−1.24 ± 0.05 ^c^	2.50 ± 0.19 ^b^	330.1 ± 1.7 ^b^	0.71 ± 0.18 ^a^
3	1	1.93 ± 0.04 ^d^	2.83 ± 0.13 ^b^	−1.78 ± 0.06 ^c^	3.35 ± 0.13 ^b^	327.7 ± 1.2 ^b^	—
3	2	2.61 ± 0.05 ^b^	3.08 ± 0.04 ^b^	−2.00 ± 0.04 ^c^	3.67 ± 0.03 ^b^	327.0 ± 0.9 ^b^	0.75 ± 0.05 ^b^
3	3	1.53 ± 0.06 ^f^	2.62 ± 0.08 ^c^	−1.39 ± 0.07 ^a^	2.96 ± 0.10 ^c^	332.1 ± 0.9 ^a^	0.61 ± 0.08 ^b^
3	4	2.23 ± 0.02 ^c^	2.76 ± 0.14 ^b^	−2.02 ± 0.14 ^c^	3.42 ± 0.16 ^b^	323.8 ± 1.6 ^b^	0.41 ± 0.09 ^b^
3	5	1.99 ± 0.10 ^d^	2.61 ± 0.05 ^c^	−2.07 ± 0.03 ^d^	3.33 ± 0.05 ^b^	321.5 ± 0.7 ^b^	0.38 ± 0.05 ^c^
3	10	2.11 ± 0.05 ^c^	2.66 ± 0.08 ^b^	−1.86 ± 0.06 ^c^	3.26 ± 0.09 ^b^	324.8 ± 0.5 ^b^	0.28 ± 0.02 ^c^
4	1	2.24 ± 0.06 ^c^	2.66 ± 0.64 ^b^	−2.60 ± 0.18 ^b^	3.28 ± 0.18 ^c^	305.9 ± 1.9 ^d^	—
4	2	2.24 ± 0.07 ^c^	2.35 ± 0.59 ^b^	−3.17 ± 0.58 ^c^	3.48 ± 0.17 ^b^	305.5 ± 2.2 ^d^	0.89 ± 0.37 ^c^
4	3	1.71 ± 0.06 ^d^	2.01 ± 0.12 ^b^	−2.57 ± 0.07 ^b^	3.26 ± 0.10 ^c^	308.0 ± 1.6 ^d^	0.84 ± 0.06 ^c^
4	4	2.84 ± 0.04 ^b^	2.15 ± 0.13 ^b^	−3.25 ± 0.08 ^c^	3.90 ± 0.02 ^b^	303.5 ± 2.2 ^d^	1.02 ± 0.09 ^c^
4	5	2.34 ± 0.05 ^c^	2.02 ± 0.17 ^b^	−3.10 ± 0.09 ^c^	3.70 ± 0.16 ^b^	303.0 ± 1.5 ^e^	0.84 ± 0.08 ^c^
4	10	2.08 ± 0.03 ^c^	2.04 ± 0.11 ^b^	−3.25 ± 0.40 ^c^	3.84 ± 0.36 ^b^	305.1 ± 2.0 ^d^	0.95 ± 0.27 ^c^
5	1	2.14 ± 0.08 ^d^	1.36 ± 0.03 ^b^	−2.32 ± 0.03 ^c^	2.69 ± 0.03 ^c^	300.4 ± 0.8 ^b^	—
5	2	2.08 ± 0.05 ^d^	1.30 ± 0.18 ^b^	−2.29 ± 0.00 ^c^	2.59 ± 0.05 ^c^	297.7 ± 2.0 ^b^	0.16 ± 0.09 ^e^
5	3	1.63 ± 0.05 ^f^	1.10 ± 0.10 ^b^	−1.97 ± 0.08 ^a^	2.26 ± 0.12 ^d^	297.2 ± 4.4 ^b^	0.68 ± 0.11 ^c^
5	4	2.40 ± 0.08 ^c^	1.20 ± 0.03 ^b^	−2.48 ± 0.09 ^d^	2.77 ± 0.08 ^c^	295.6 ± 0.6 ^b^	0.35 ± 0.10 ^d^
5	5	1.89 ± 0.04 ^e^	1.08 ± 0.10 ^b^	−2.23 ± 0.04 ^c^	2.48 ± 0.03 ^d^	295.8 ± 2.3 ^b^	0.40 ± 0.05 ^d^
5	10	1.94 ± 0.04 ^e^	1.25 ± 0.31 ^b^	−2.36 ± 0.03 ^c^	2.60 ± 0.03 ^c^	294.5 ± 0.8 ^b^	0.35 ± 0.02 ^d^
6	1	1.64 ± 0.07 ^e^	0.90 ± 0.08 ^c^	−2.06 ± 0.04 ^b^	2.25 ± 0.04 ^c^	293.7 ± 2.0 ^c^	—
6	2	1.71 ± 0.03 ^e^	0.66 ± 0.14 ^c^	−2.14 ± 0.04 ^b^	2.24 ± 0.04 ^c^	287.2 ± 3.4 ^d^	0.27 ± 0.13 ^c^
6	3	1.27 ± 0.07 ^g^	0.73 ± 0.03 ^c^	−1.72 ± 0.02 ^a^	1.87 ± 0.03 ^d^	293.0 ± 0.6 ^c^	0.53 ± 0.05 ^c^
6	4	2.14 ± 0.03 ^d^	0.86 ± 0.08 ^c^	−2.17 ± 0.03 ^b^	2.33 ± 0.03 ^c^	291.5 ± 2.1 ^c^	0.53 ± 0.02 ^c^
6	5	1.83 ± 0.03 ^e^	0.50 ± 0.13 ^c^	−2.00 ± 0.03 ^b^	2.07 ± 0.05 ^c^	284.4 ± 3.7 ^d^	0.45 ± 0.12 ^c^
6	10	1.98 ± 0.03 ^d^	0.61 ± 0.02 ^c^	−2.12 ± 0.04 ^b^	2.29 ± 0.05 ^c^	286.0 ± 0.5 ^d^	0.46 ± 0.02 ^c^
7	1	1.56 ± 0.07 ^e^	0.50 ± 0.04 ^c^	−2.05 ± 0.13 ^b^	2.11 ± 0.14 ^d^	283.7 ± 0.3 ^b^	—
7	2	1.58 ± 0.03 ^e^	0.47 ± 0.07 ^c^	−2.06 ± 0.05 ^b^	2.12 ± 0.03 ^c^	282.9 ± 2.2 ^b^	0.07 ± 0.05 ^e^
7	3	1.14 ± 0.07 ^f^	0.44 ± 0.09 ^c^	−1.63 ± 0.10 ^a^	1.68 ± 0.11 ^e^	284.4 ± 2.1 ^b^	0.61 ± 0.11 ^c^
7	4	2.21 ± 0.03 ^c^	0.48 ± 0.06 ^c^	−2.34 ± 0.09 ^c^	2.39 ± 0.08 ^c^	281.6 ± 1.8 ^c^	0.72 ± 0.06 ^c^
7	5	1.86 ± 0.03 ^d^	0.38 ± 0.15 ^c^	−1.99 ± 0.05 ^b^	2.04 ± 0.07 ^d^	281.2 ± 4.5 ^c^	0.34 ± 0.08 ^d^
7	10	2.14 ± 0.03 ^c^	0.49 ± 0.01 ^c^	−2.22 ± 0.09 ^c^	2.27 ± 0.08 ^c^	282.3 ± 0.6 ^b^	0.61 ± 0.05 ^c^
8	1	1.58 ± 0.06 ^d^	0.24 ± 0.17 ^b^	−1.93 ± 0.07 ^b^	1.95 ± 0.09 ^b^	277.1 ± 4.7 ^b^	—
8	2	1.59 ± 0.06 ^d^	0.31 ± 0.10 ^b^	−1.96 ± 0.03 ^b^	1.99 ± 0.04 ^b^	281.0 ± 6.2 ^b^	0.11 ± 0.07 ^c^
8	3	1.10 ± 0.02 ^f^	0.12 ± 0.09 ^b^	−1.47 ± 0.09 ^a^	1.48 ± 0.08 ^c^	274.7 ± 3.4 ^b^	0.68 ± 0.05 ^b^
8	4	1.99 ± 0.13 ^c^	0.12 ± 0.05 ^b^	−2.19 ± 0.32 ^c^	2.20 ± 0.32 ^b^	273.3 ± 1.9 ^b^	0.54 ± 0.27 ^b^
8	5	1.73 ± 0.02 ^d^	0.10 ± 0.03 ^b^	−1.81 ± 0.09 ^b^	1.81 ± 0.10 ^c^	270.5 ± 4.5 ^b^	0.24 ± 0.08 ^c^
8	10	1.94 ± 0.03 ^c^	−0.05 ± 0.06 ^c^	−1.67 ± 0.12 ^b^	1.67 ± 0.12 ^c^	267.5 ± 2.2 ^c^	0.54 ± 0.09 ^b^
9	1	1.53 ± 0.09 ^c^	−0.31 ± 0.17 ^a^	−1.21 ± 0.05 ^d^	1.26 ± 0.01 ^a^	256.0 ± 7.9 ^a^	—
9	2	1.72 ± 0.02 ^c^	−0.49 ± 0.11 ^a^	−1.08 ± 0.12 ^d^	1.19 ± 0.12 ^a^	245.6 ± 5.6 ^a^	0.31 ± 0.11 ^d^
9	3	1.60 ± 0.06 ^c^	−0.69 ± 0.14 ^b^	−0.47 ± 0.09 ^b^	0.86 ± 0.07 ^b^	214.3 ± 9.5 ^c^	0.85 ± 0.12 ^c^
9	4	2.70 ± 0.64 ^a^	−0.95 ± 0.07 ^b^	−0.52 ± 0.10 ^b^	1.09 ± 0.03 ^b^	208.7 ± 6.3 ^c^	1.52 ± 0.58 ^b^
9	5	2.50 ± 0.15 ^a^	−0.95 ± 0.09 ^b^	−0.26 ± 0.08 ^b^	0.99 ± 0.09 ^b^	193.5 ± 1.4 ^d^	1.51 ± 0.06 ^b^
9	10	2.97 ± 0.01 ^a^	−1.00 ± 0.06 ^c^	0.20 ± 0.04 ^a^	1.02 ± 0.06 ^b^	168.6 ± 1.5 ^e^	2.13 ± 0.04 ^a^
10	1	1.90 ± 0.04 ^c^	−0.83 ± 0.07 ^b^	−0.62 ± 0.07 ^c^	1.04 ± 0.02 ^a^	216.9 ± 5.4 ^a^	—
10	2	2.47 ± 0.01 ^b^	−0.62 ± 0.12 ^a^	−0.09 ± 0.05 ^b^	0.62 ± 0.12 ^b^	188.9 ± 5.3 ^b^	0.81 ± 0.04 ^a^
10	3	2.18 ± 0.03 ^b^	−0.61 ± 0.02 ^a^	0.32 ± 0.17 ^a^	0.65 ± 0.04 ^b^	161.6 ± 6.0 ^c^	1.00 ± 0.16 ^a^
10	4	2.69 ± 0.04 ^a^	−0.61 ± 0.10 ^a^	−0.06 ± 0.10 ^b^	0.62 ± 0.10 ^b^	186.0 ± 8.5 ^b^	1.00 ± 0.04 ^a^
10	5	2.47 ± 0.04 ^b^	−0.51 ± 0.09 ^a^	0.07 ± 0.26 ^a^	0.53 ± 0.07 ^b^	193.8 ± 11.9 ^b^	0.97 ± 0.12 ^a^
10	10	2.66 ± 0.06 ^a^	−0.40 ± 0.10 ^a^	−0.30 ± 0.04 ^b^	0.51 ± 0.07 ^b^	217.0 ± 8.4 ^a^	0.94 ± 0.09 ^a^

Values are expressed as mean ± standard deviation (*n* = 3). L* = lightness; a* and b* = color coordinates; C* = chroma; h = hue angle. The total color difference (ΔE) was calculated using the values from day 1 as a reference. Statistically significant differences were assessed using one-way ANOVA, followed by the Tukey HSD test (*p* < 0.05). Different letters indicate statistically significant differences.

**Table 3:** Colorimetric characterization (CIELAB) of aqueous extracts at different pH values.

pH	Day	L*	a*	b*	C*	h	ΔE
1	1	1.68 ± 0.02 ^b^	1.55 ± 0.13 ^b^	−0.94 ± 0.10 ^b^	1.82 ± 0.07 ^b^	328.7 ± 4.7 ^c^	—
1	2	1.98 ± 0.06 ^a^	1.97 ± 0.14 ^b^	−1.15 ± 0.17 ^c^	2.30 ± 0.19 ^b^	330.1 ± 3.2 ^c^	0.58 ± 0.13 ^a^
1	3	1.07 ± 0.07 ^b^	1.68 ± 0.07 ^b^	−0.80 ± 0.09 ^b^	1.87 ± 0.04 ^b^	334.6 ± 3.2 ^c^	0.65 ± 0.04 ^a^
1	4	1.70 ± 0.07 ^b^	1.83 ± 0.09 ^b^	−1.08 ± 0.05 ^c^	2.13 ± 0.04 ^b^	331.0 ± 0.4 ^c^	0.32 ± 0.05 ^a^
1	5	1.75 ± 0.02 ^b^	1.87 ± 0.05 ^b^	−1.31 ± 0.06 ^c^	2.29 ± 0.02 ^b^	324.9 ± 1.9 ^d^	0.50 ± 0.02 ^a^
1	10	1.82 ± 0.08 ^a^	1.77 ± 0.09 ^b^	−1.40 ± 0.14 ^d^	2.28 ± 0.04 ^b^	321.7 ± 3.6 ^d^	0.54 ± 0.07 ^a^
2	1	1.55 ± 0.07 ^b^	1.36 ± 0.13 ^c^	−0.98 ± 0.08 ^b^	1.68 ± 0.13 ^c^	324.2 ± 2.6 ^b^	—
2	2	2.03 ± 0.07 ^a^	1.55 ± 0.08 ^c^	−1.22 ± 0.11 ^c^	2.01 ± 0.06 ^c^	322.0 ± 4.7 ^c^	0.57 ± 0.04 ^a^
2	3	1.22 ± 0.06 ^b^	1.68 ± 0.10 ^c^	−0.99 ± 0.15 ^b^	1.95 ± 0.16 ^c^	329.8 ± 2.4 ^b^	0.48 ± 0.11 ^b^
2	4	2.04 ± 0.06 ^a^	1.64 ± 0.05 ^c^	−1.58 ± 0.07 ^d^	2.28 ± 0.06 ^b^	316.1 ± 1.5 ^c^	0.82 ± 0.03 ^a^
2	5	1.68 ± 0.05 ^a^	1.62 ± 0.07 ^c^	−1.43 ± 0.02 ^c^	2.16 ± 0.05 ^b^	318.7 ± 1.5 ^c^	0.53 ± 0.02 ^b^
2	10	1.89 ± 0.05 ^a^	1.71 ± 0.15 ^c^	−1.61 ± 0.06 ^d^	2.34 ± 0.09 ^b^	320.0 ± 8.1 ^c^	0.80 ± 0.03 ^a^
3	1	1.74 ± 0.02 ^e^	1.66 ± 0.12 ^d^	−1.51 ± 0.07 ^b^	2.25 ± 0.06 ^d^	317.5 ± 3.4 ^c^	—
3	2	2.12 ± 0.07 ^c^	1.67 ± 0.07 ^d^	−1.64 ± 0.01 ^b^	2.35 ± 0.04 ^d^	315.4 ± 1.2 ^c^	0.41 ± 0.06 ^b^
3	3	1.29 ± 0.03 ^g^	1.40 ± 0.03 ^d^	−1.23 ± 0.08 ^a^	1.86 ± 0.04 ^e^	318.8 ± 2.3 ^c^	0.60 ± 0.05 ^b^
3	4	2.18 ± 0.06 ^c^	1.66 ± 0.12 ^d^	−1.75 ± 0.08 ^b^	2.41 ± 0.13 ^d^	313.8 ± 0.9 ^c^	0.51 ± 0.02 ^b^
3	5	1.98 ± 0.10 ^d^	1.59 ± 0.11 ^d^	−1.89 ± 0.12 ^c^	2.47 ± 0.17 ^d^	307.0 ± 5.7 ^d^	0.47 ± 0.04 ^b^
3	10	4.97 ± 0.11 ^a^	8.53 ± 0.40 ^a^	−3.73 ± 0.18 ^e^	9.28 ± 0.44 ^a^	337.2 ± 0.9 ^a^	7.91 ± 0.34 ^a^
4	1	1.56 ± 0.35 ^d^	1.48 ± 0.15 ^c^	−1.52 ± 0.12 ^a^	2.13 ± 0.16 ^d^	314.1 ± 2.7 ^c^	—
4	2	2.18 ± 0.11 ^c^	1.64 ± 0.02 ^c^	−1.69 ± 0.04 ^a^	2.34 ± 0.04 ^d^	313.9 ± 0.2 ^c^	0.66 ± 0.10 ^c^
4	3	1.42 ± 0.14 ^d^	1.69 ± 0.17 ^c^	−1.39 ± 0.08 ^a^	2.19 ± 0.18 ^d^	320.5 ± 1.2 ^b^	0.33 ± 0.08 ^d^
4	4	2.29 ± 0.03 ^c^	1.63 ± 0.11 ^c^	−1.92 ± 0.10 ^a^	2.52 ± 0.14 ^d^	310.2 ± 1.3 ^c^	0.85 ± 0.07 ^c^
4	5	2.51 ± 0.05 ^b^	2.80 ± 0.06 ^b^	−2.16 ± 0.06 ^b^	3.54 ± 0.08 ^b^	322.4 ± 0.3 ^b^	1.75 ± 0.04 ^b^
4	10	7.07 ± 0.11 ^a^	13.65 ± 0.16 ^a^	−4.82 ± 0.03 ^d^	14.47 ± 0.14 ^a^	340.5 ± 0.3 ^a^	13.76 ± 0.17 ^a^
5	1	1.99 ± 0.01 ^d^	0.53 ± 0.09 ^c^	−1.75 ± 0.10 ^a^	1.84 ± 0.14 ^f^	287.4 ± 2.6 ^c^	—
5	2	2.32 ± 0.02 ^c^	0.69 ± 0.03 ^c^	−1.99 ± 0.14 ^b^	2.11 ± 0.13 ^e^	288.1 ± 3.3 ^c^	0.44 ± 0.10 ^d^
5	3	1.59 ± 0.03 ^f^	0.72 ± 0.16 ^c^	−1.79 ± 0.11 ^a^	1.92 ± 0.10 ^e^	291.9 ± 3.9 ^c^	0.47 ± 0.07 ^d^
5	4	2.43 ± 0.05 ^c^	0.78 ± 0.05 ^c^	−2.26 ± 0.06 ^c^	2.39 ± 0.08 ^d^	289.0 ± 0.6 ^c^	0.72 ± 0.09 ^c^
5	5	5.20 ± 0.16 ^b^	5.24 ± 0.04 ^a^	−5.73 ± 0.09 ^e^	7.77 ± 0.08 ^b^	312.5 ± 0.3 ^a^	6.95 ± 0.04 ^b^
5	10	10.38 ± 0.03 ^a^	5.14 ± 0.02 ^a^	−7.09 ± 0.02 ^f^	8.74 ± 0.03 ^a^	305.9 ± 0.1 ^a^	10.96 ± 0.01 ^a^
6	1	1.98 ± 0.08 ^d^	0.68 ± 0.09 ^c^	−1.82 ± 0.10 ^a^	1.94 ± 0.12 ^d^	290.3 ± 1.6 ^c^	—
6	2	2.33 ± 0.03 ^c^	0.69 ± 0.08 ^c^	−1.86 ± 0.04 ^a^	1.98 ± 0.06 ^d^	290.0 ± 2.1 ^c^	0.36 ± 0.03 ^c^
6	3	1.56 ± 0.10 ^f^	0.70 ± 0.07 ^c^	−1.64 ± 0.13 ^a^	1.76 ± 0.07 ^d^	293.2 ± 3.4 ^c^	0.49 ± 0.03 ^c^
6	4	2.42 ± 0.06 ^c^	0.74 ± 0.08 ^c^	−2.17 ± 0.12 ^b^	2.29 ± 0.14 ^c^	288.7 ± 1.0 ^c^	0.57 ± 0.09 ^c^
6	5	3.51 ± 0.10 ^b^	3.56 ± 0.13 ^b^	−3.88 ± 0.28 ^c^	5.30 ± 0.18 ^b^	311.0 ± 1.0 ^b^	3.86 ± 0.21 ^b^
6	10	7.22 ± 0.13 ^a^	8.99 ± 0.57 ^a^	−6.85 ± 0.08 ^d^	11.59 ± 0.11 ^a^	323.6 ± 0.3 ^a^	11.04 ± 0.45 ^a^
7	1	1.80 ± 0.04 ^d^	0.46 ± 0.10 ^c^	−1.81 ± 0.08 ^b^	1.87 ± 0.09 ^d^	284.1 ± 2.3 ^b^	—
7	2	2.34 ± 0.04 ^c^	0.34 ± 0.01 ^c^	−1.93 ± 0.14 ^b^	1.96 ± 0.14 ^d^	280.1 ± 0.8 ^c^	0.58 ± 0.03 ^c^
7	3	1.35 ± 0.01 ^e^	0.34 ± 0.11 ^c^	−1.46 ± 0.07 ^a^	1.50 ± 0.06 ^e^	283.9 ± 5.0 ^b^	0.59 ± 0.05 ^c^
7	4	2.25 ± 0.07 ^c^	0.35 ± 0.12 ^c^	−2.01 ± 0.11 ^b^	2.04 ± 0.13 ^d^	279.5 ± 2.2 ^c^	0.52 ± 0.03 ^c^
7	5	3.80 ± 0.25 ^b^	1.48 ± 0.04 ^b^	−4.32 ± 0.02 ^d^	4.57 ± 0.03 ^b^	288.9 ± 0.4 ^b^	3.37 ± 0.15 ^b^
7	10	10.26 ± 0.09 ^a^	6.13 ± 0.08 ^a^	−6.87 ± 0.05 ^e^	9.21 ± 0.08 ^a^	311.7 ± 0.4 ^a^	11.37 ± 0.12 ^a^
8	1	1.74 ± 0.04 ^d^	−0.05 ± 0.04 ^c^	−1.58 ± 0.03 ^a^	1.58 ± 0.03 ^c^	268.2 ± 1.4 ^c^	—
8	2	2.33 ± 0.05 ^b^	−0.25 ± 0.12 ^c^	−1.72 ± 0.13 ^b^	1.73 ± 0.12 ^c^	261.6 ± 4.2 ^c^	0.66 ± 0.03 ^b^
8	3	1.30 ± 0.02 ^e^	−0.05 ± 0.14 ^c^	−1.29 ± 0.07 ^a^	1.30 ± 0.08 ^d^	266.2 ± 3.5 ^c^	0.54 ± 0.02 ^b^
8	4	2.25 ± 0.05 ^b^	−0.22 ± 0.05 ^c^	−1.79 ± 0.03 ^b^	1.80 ± 0.03 ^c^	262.4 ± 2.2 ^c^	0.58 ± 0.04 ^b^
8	5	2.24 ± 0.04 ^b^	−0.35 ± 0.15 ^d^	−1.63 ± 0.10 ^a^	1.67 ± 0.07 ^c^	257.9 ± 5.8 ^c^	0.60 ± 0.07 ^b^
8	10	11.51 ± 0.10 ^a^	1.27 ± 0.05 ^a^	−2.93 ± 0.03 ^d^	3.19 ± 0.03 ^a^	292.9 ± 0.5 ^a^	9.95 ± 0.11 ^a^
9	1	1.66 ± 0.03 ^c^	−0.32 ± 0.05 ^a^	−1.34 ± 0.18 ^e^	1.38 ± 0.17 ^a^	256.1 ± 3.5 ^a^	—
9	2	2.62 ± 0.04 ^a^	−0.42 ± 0.08 ^a^	−1.02 ± 0.08 ^d^	1.10 ± 0.06 ^b^	247.6 ± 5.1 ^a^	1.02 ± 0.02 ^b^
9	3	1.73 ± 0.05 ^c^	−0.38 ± 0.06 ^a^	−0.48 ± 0.04 ^b^	0.61 ± 0.01 ^c^	231.9 ± 6.0 ^b^	0.86 ± 0.04 ^c^
9	4	2.32 ± 0.12 ^b^	−0.34 ± 0.08 ^a^	−0.75 ± 0.10 ^c^	0.83 ± 0.06 ^c^	246.0 ± 6.2 ^a^	0.90 ± 0.04 ^c^
9	5	2.77 ± 0.09 ^a^	−0.52 ± 0.12 ^a^	−0.69 ± 0.11 ^c^	0.88 ± 0.06 ^b^	232.8 ± 9.6 ^b^	1.31 ± 0.13 ^b^
9	10	3.02 ± 0.04 ^a^	−0.56 ± 0.02 ^a^	−0.43 ± 0.07 ^b^	0.61 ± 0.14 ^c^	216.5 ± 5.1 ^b^	1.65 ± 0.02 ^a^
10	1	2.11 ± 0.04 ^b^	−0.50 ± 0.02 ^a^	−0.59 ± 0.10 ^c^	0.77 ± 0.06 ^b^	230.0 ± 6.9 ^a^	—
10	2	3.20 ± 0.17 ^a^	−0.37 ± 0.02 ^a^	−0.30 ± 0.16 ^b^	0.49 ± 0.10 ^c^	216.5 ± 13.7 ^a^	1.14 ± 0.13 ^a^
10	3	2.23 ± 0.70 ^b^	−0.49 ± 0.22 ^a^	−0.17 ± 0.10 ^b^	0.53 ± 0.20 ^b^	202.3 ± 18.5 ^b^	0.71 ± 0.25 ^b^
10	4	2.30 ± 0.08 ^b^	−0.33 ± 0.17 ^a^	−0.49 ± 0.03 ^c^	0.61 ± 0.08 ^b^	237.1 ± 14.9 ^a^	0.29 ± 0.15 ^c^
10	5	2.56 ± 0.03 ^b^	−0.39 ± 0.10 ^a^	−0.52 ± 0.03 ^c^	0.66 ± 0.03 ^b^	234.1 ± 8.5 ^a^	0.48 ± 0.04 ^b^
10	10	2.91 ± 0.04 ^a^	−0.32 ± 0.20 ^a^	−0.59 ± 0.13 ^c^	0.70 ± 0.05 ^b^	241.6 ± 19.2 ^a^	0.84 ± 0.03 ^a^

Values are expressed as mean ± standard deviation (*n* = 3). L* = lightness; a* and b* = color coordinates; C* = chroma; h = hue angle. The total color difference (ΔE) was calculated using the values from day 1 as a reference. Statistically significant differences were assessed using one-way ANOVA, followed by the Tukey HSD test (*p* < 0.05). Different letters indicate statistically significant differences.

**Table 4:** Microbiological characterization of red cabbage extracts.

Sample Code		Type of Analysis				
		REB	REC	RCC	YM	NTG
					CFU/g	CFU/g
P1	Day 1	Absent	Absent	Absent	0	0
	Day 5	Absent	Absent	Absent	0	100
	Day 10	Absent	Absent	Absent	200	100
P2	Day 1	Absent	Absent	Absent	0	0
	Day 5	Absent	Absent	Absent	100	0
	Day 10	Absent	Absent	Absent	400	100
P3	Day 1	Absent	Absent	Absent	0	0
	Day 5	Absent	Absent	Absent	0	0
	Day 10	Absent	Absent	Absent	100	200
P4	Day 1	Absent	Absent	Absent	0	0
	Day 5	Absent	Absent	Absent	0	0
	Day 10	Absent	Absent	Absent	0	300
P5	Day 1	Absent	Absent	Absent	0	0
	Day 5	Absent	Absent	Absent	0	100
	Day 10	Absent	Absent	Absent	300	100
P6	Day 1	Absent	Absent	Absent	0	0
	Day 5	Absent	Absent	Absent	100	200
	Day 10	Absent	Absent	Absent	300	200
P7	Day 1	Absent	Absent	Absent	0	0
	Day 5	Absent	Absent	Absent	200	200
	Day 10	Absent	Absent	Absent	200	400
P8	Day 1	Absent	Absent	Absent	0	0
	Day 5	Absent	Absent	Absent	200	0
	Day 10	Absent	Absent	Absent	600	200
P9	Day 1	Absent	Absent	Absent	0	100
	Day 5	Absent	Absent	Absent	300	100
	Day 10	Absent	Absent	Absent	500	500
P10	Day 1	Absent	Absent	Absent	0	0
	Day 5	Absent	Absent	Absent	100	300
	Day 10	Absent	Absent	Absent	300	600
P11	Day 1	Absent	Absent	Absent	0	0
	Day 5	Absent	Absent	Absent	200	300
	Day 10	Absent	Absent	Absent	200	500
P12	Day 1	Absent	Absent	Absent	100	0
	Day 5	Absent	Absent	Absent	300	100
	Day 10	Absent	Absent	Absent	700	600
P13	Day 1	Absent	Absent	Absent	100	100
	Day 5	Absent	Absent	Absent	600	300
	Day 10	Absent	Absent	Absent	1000	500
P14	Day 1	Absent	Absent	Absent	200	100
	Day 5	Absent	Absent	Absent	600	300
	Day 10	Absent	Absent	Absent	900	800
P15	Day 1	Absent	Absent	Absent	100	100
	Day 5	Absent	Absent	Absent	300	400
	Day 10	Absent	Absent	Absent	1300	600
P16	Day 1	Absent	Absent	Absent	100	0
	Day 5	Absent	Absent	Absent	300	500
	Day 10	Absent	Absent	Absent	1100	900

**Table 5:** Sample coding and experimental storage conditions used to evaluate the stability of anthocyanin extracts from red cabbage.

Sample Name	Varied Parameters			
	Light/Dark	Oxygenation Mode	Temperature	Various Extraction Solvents
P1	Light	Aerobic (O_2_ present)	4 ± 0.5 °C	50% ethanol (*v*/*v*)
P2	Dark	Aerobic (O_2_ present)	4 ± 0.5 °C	50% ethanol (*v*/*v*)
P3	Light	Anaerobic (O_2_ absent)	4 ± 0.5 °C	50% ethanol (*v*/*v*)
P4	Dark	Anaerobic (O_2_ absent)	4 ± 0.5 °C	50% ethanol (*v*/*v*)
P5	Light	Aerobic (O_2_ present)	25 ± 0.5 °C	50% ethanol (*v*/*v*)
P6	Dark	Aerobic (O_2_ present)	25 ± 0.5 °C	50% ethanol (*v*/*v*)
P7	Light	Anaerobic (O_2_ absent)	25 ± 0.5 °C	50% ethanol (*v*/*v*)
P8	Dark	Anaerobic (O_2_ absent)	25 ± 0.5 °C	50% ethanol (*v*/*v*)
P9	Light	Aerobic (O_2_ present)	4 ± 0.5 °C	Water
P10	Dark	Aerobic (O_2_ present)	4 ± 0.5 °C	Water
P11	Light	Anaerobic (O_2_ absent)	4 ± 0.5 °C	Water
P12	Dark	Anaerobic (O_2_ absent)	4 ± 0.5 °C	Water
P13	Light	Aerobic (O_2_ present)	25 ± 0.5 °C	Water
P14	Dark	Aerobic (O_2_ present)	25 ± 0.5 °C	Water
P15	Light	Anaerobic (O_2_ absent)	25 ± 0.5 °C	Water
P16	Dark	Anaerobic (O_2_ absent)	25 ± 0.5 °C	Water

## Data Availability

The data presented in this study are available on request from the corresponding author.
